# Supplementary material for: Situation analysis for delivering integrated comprehensive sexual and reproductive health services for displaced population of Kasaï, Democratic Republic of Congo: Protocol for a mixed method study
Source: PLoS One. 2020 Dec 21;15(12):e0242046. doi: 10.1371/journal.pone.0242046 (PMC7751877; doi:10.1371/journal.pone.0242046)
Supplement: S1 Appendix — (DOCX) [file pone.0242046.s001.docx]

**Situation Analysis for Delivering Integrated Comprehensive SRHR Services for Kasaï displaced people in the Democratic Republic of Congo (DRC)**

**FOCUS GROUP DISCUSSION GUIDE – APPENDIX C4 (MALE)**

**Project Overview**

WHO has undertaken a multi-country project to deliver integrated comprehensive SRHR services to meet the immediate SRH needs of extremely vulnerable women, adolescent girls in acute humanitarian crises, particularly in relation to contraceptive and safe abortion services. One of the project sites is Kasaï displaced camps in the Democratic Republic of Congo (DRC). With the roll-out of Phase 1 of the Health Sector response (implementation of Minimum Initial Services Package and improving functional referral pathways through community outreach), WHO and Population and Health Research Institute (PHERI) are undertaking this research to understand the feasibility of implementing an integrated and effective package of services.

**Purpose of the research**

The purpose of this study is to understand your needs of Sexual and Reproductive Health and Rights services, SRH sufferings, health-seeking behaviors, utilization of health facilities and barriers in accessing SRH services. As you are 15-59 years’ aged Men, you are being requested to participate in this Focus Group Discussion.

**Informed Consent Form**

Focus group discussions are limited to men living within the study catchment areas. Copies of informed consent and confidentiality forms should be provided to each participant and read aloud for the benefit of those who cannot read. Participants should be provided an opportunity to ask any questions. The following is a guide. Try to ask all the questions below in the order given, but it is more important to maintain the flow of discussion. Suggested probes have been included. You should try to encourage participation of all group members in the conversation.

**What will happen if you take part in the study?**

If you decide to take part in the study, you will be asked to do the following activities:

**Face to face interview:** One interviewer will conduct face-to-face group interview with you at your convenient place. If you give permission to record the interview, then the group interview will be recorded. A total of 8/10 FGD will be conducted with men (young and adults) unmarried and married will take part at each FGD of the study.

**Risk**

There is no risk of physical or emotional harm if you participate in this study.

**Benefits**

There is no direct benefit for taking part in this study. The information to be collected through this study will help improving sexual and reproductive health care services in Rohingya Refugee Camps at Cox’s Bazar in Bangladesh.

**Privacy, anonymity and confidentiality**

Identifier information collected in this study will be coded with a number and will be kept confidential.

All information will be saved in a different encrypted file where only authorized research staff will have access. Your name or any other privacy related information will never appear in any publication or results from the study.

**Future use of information**

If there is a need for future use of the information collected by data collectors, we will provide only de-identified data so that privacy, anonymity and confidentiality of the participants are ensured.

**Right not to participate and withdraw**

Participation in this research is voluntary. You have the right to know about the procedures, risks, and benefits of the study. Even if you decide to take part, you can change your mind later and can leave the study at any time. No matter what decision you make, there will be no problems for you.

**Compensation**

There will be no financial compensation for taking part in the study.

**Answering your questions/ Contact persons**

If you have any questions about this research project please contact Mr Fidèle Mbadu (+243898940247) or Prof. Jacques B.O. Emina (+243814092128).

If you agree to take part in our study, please indicate that by putting your signature or your left thumb impression at the specified space below.

Thank you for your cooperation.

| Name of the participants | Signature or left thumb impression of participant |
| --- | --- |
|  |  |
|  |  |
|  |  |
|  |  |
|  |  |
|  |  |
|  |  |
|  |  |
|  |  |
|  |  |

| Date: _______________________________ | |
| --- | --- |
| Starting time: _____________________________ | Ending time: __________________________ |
| Interviewer’s Name  Interviewer’s Signature  Note taker’s Name  Note taker’s Signature  Supervisor’s Signature | _____________________________________  _____________________________________  _____________________________________  _____________________________________  _____________________________________ |

**Start by explaining the ground rules as follows**

Before we start I would like to remind you that there are no right or wrong answers in this discussion. We are interested in knowing what each of you think, so please feel free to be frank and to share your point of view, regardless of whether you agree or disagree with what you hear. It is very important that we hear all your opinions. You probably prefer that your comments not be repeated to people outside this group. Please treat others in the group as you want to be treated by not telling anyone about what you hear in this discussion today. Let's start by going around the circle and having each person introduce himself.

**Section 1: Socio-economic characteristics of the respondent (please fill up for all of them)**

| Respondent name/ID | Age | Religion | Marital status | Education | Profession |
| --- | --- | --- | --- | --- | --- |
|  |  |  |  |  |  |
|  |  |  |  |  |  |
|  |  |  |  |  |  |
|  |  |  |  |  |  |
|  |  |  |  |  |  |
|  |  |  |  |  |  |

**SRH service needs and utilization**

- What are the most common SRH related health issues among Kasaï displaced people in this camp/ village?

*Please be specific and details as much as possible*.

- - For women
  - For men
  - For adolescent girls
  - For adolescent boys

Ask separately for Family Planning, delivery, maternal care, Condom, HIV related services (testing and care), Gender-based violence, law to protect women and girl.

- Do you perceive SRH issues [Family Planning, delivery, maternal care, Condom, HIV related services (testing and care), Gender-based violence] for girls/women are different from boys/men? How so? Please explain?

**Health seeking Behaviors (Current practice)**

- For any specific SRH issue ( for exp. menstrual health, Pregnancy and delivery care, family planning and contraception use, abortion/MR, STD/STI) who do you perceive the community should seek advice from?

*Probe Why? If they failed then what they do? Where do they go for seeking healthcare services? If they do not seek any healthcare service, then why not? Ask separately for,*

- - For women
  - For men
  - For adolescent girls
  - For adolescent boys

**Available SRH related services in the camp/area**

- What are the main health care facilities that your community can access in this camp/ area? Please be specific. (Probe: what are their services and for whom. How services are provided?)
- Do you think, the healthcare facilities are sufficient enough to meet the SRH needs of the community at large in this area? If not, Why? Please explain.

Ask separately for,

- - For women
  - For men
  - For adolescent girls
  - For adolescent boys

Ask separately for Family Planning, delivery, maternal care, Condom, HIV related services (testing and care).

- What other services do you think are important to provide to meet the SRH needs of the Kasaï displaced people living in this camp/ village?
  - For women
  - for men
  - for adolescent girls
  - for adolescent boys

Ask separately for Family Planning, delivery, maternal care, Condom, HIV related services (testing and care)

- In your opinion, do you think if all the services you have mentioned are provided will be life saving for your community people and should be prioritized (yes/no – why probe) and how so?

**Ranking of SRH Needs**

- According to the response of the participants, ask the participants to rank the most five immediate needs of the Kasaï displaced people staying in this camp? Why?

Ask separately for,

- - For women
  - For men
  - For adolescent girls
  - For adolescent boys

Ask separately for Family Planning, delivery, maternal care, Condom, HIV related services (testing and care).

**Knowledge and awareness about SRH service provider**

- Do you know which organization/ health centers in this camp/ area are providing SRHR related services only? (Probe: what are their services and for whom. How services are provided?)

Ask separately for,

- - For women
  - For men
  - For adolescent girls
  - For adolescent boys

Ask separately for Family Planning, delivery, maternal care, Condom, HIV related services (testing and care)

**Discussion on SRH issues**

- How do you feel about husbands discussing SRH (FP, HIV/AIDS, gender-based violence, etc) issues with their wives?
- How do you feel parents discussing SRH (FP, HIV/AIDS, gender-based violence, etc) issues with their children?
- How do you feel sibling discussing SRH (FP, HIV/AIDS, gender-based violence, etc) issues?
- How do you feel SRH (FP, HIV/AIDS, gender-based violence, etc) issues being taught at school?
- How do you feel adolescents and young people seeking SRH (FP, HIV/AIDS, gender-based violence, etc) services?

**Utilization of health care facilities and barriers in accessing SRH service**

- Do you think, the Kasaï displaced people face any challenges while seeking access to SRH services in this camp? If yes, what are the challenges and barriers in accessing available services?

Ask separately for ,

- - For women
  - For men
  - For adolescent girls
  - For adolescent boys

Ask separately for Family Planning, delivery, maternal care, Condom, HIV related services (testing and care)

- Are these challenges in service access different for men compared to women, if so please explain how?
- Are these challenges in service access different for boys compared to girls, if so please explain how?
- Please recommend, how the access and utilization of healthcare facilities can be increased.
