# Supplementary material for: Situation analysis for delivering integrated comprehensive sexual and reproductive health services for displaced population of Kasaï, Democratic Republic of Congo: Protocol for a mixed method study
Source: PLoS One. 2020 Dec 21;15(12):e0242046. doi: 10.1371/journal.pone.0242046 (PMC7751877; doi:10.1371/journal.pone.0242046)
Supplement: S1 Annex — (ZIP) [file pone.0242046.s002.zip › Annex A1-Household_QRE_EN_MARCH2020_A1.pdf]

SITUATION ANALYSIS FOR DELIVERING INTEGRATED COMPREHENSIVE SEXUAL AND REPRODUCTIVE HEALTH SERVICES FOR DISPLACED POPULATION OF KASAI DEMOCRATIC REPUBLIC OF CONGO (DRC)

HOUSEHOLD QUESTIONNAIRE

PHERI-PNSA - WHO

IDENTIFICATION

VILLAGE/ CAMP NAME ______________________ [ ][ ]

SPECIFY (VILLAGE=1 OR DISPLACED CAMP=2) [ ]

DO YOU ALWAYS LIVE IN THIS VILLAGE
1. YES, I AM NATIVE OF THIS VILLAGE (END OF INTERVIEW)
2. I HAVE BEEN LIVING THERE FOR SEVERAL YEARS/ BEFORE THE CONFLICT (END OF INTERVIEW)
3. I AM ARRIVING HERE FLEEING FROM THE KAMUINA NSAPU CONFLICT

[ ]

NAME OF HOUSEHOLD HEAD ______________________

HEALTH ZONE ______________________ [ ][ ]

PROVINCE . . . . [ ]

HOUSEHOLD NUMBER . . . . [ ][ ][ ][ ]

INTERVIEWER VISITS

	1	2	3	FINAL VISIT
DATE	________	________	________	DAY [ ][ ]<br>MONTH [ ][ ]<br>YEAR [ ][ ][ ][ ]
INTERVIEWER'S NAME	________	________	________	INT. NO. [ ][ ][ ][ ]
RESULT*	________	________	________	RESULT* [ ]
NEXT VISITDATE	________	________		TOTAL NUMBER OF VISITS [ ]
TIME	________	________		

*RESULT CODES:

1 COMPLETED
2 NO HOUSEHOLD MEMBER AT HOME OR NO COMPETENT RESPONDENT AT HOME AT TIME OF VISIT
3 ENTIRE HOUSEHOLD ABSENT FOR EXTENDED PERIOD OF TIME
4 POSTPONED
5 REFUSED
6 DWELLING VACANT OR ADDRESS NOT A DWELLING
7 DWELLING DESTROYED
8 DWELLING NOT FOUND
9 OTHER ______________________
(SPECIFY)

TOTAL PERSONS IN HOUSEHOLD [ ][ ]

TOTAL ELIGIBLE WOMEN [ ][ ]

LINE NO. OF RESPONDENT TO HOUSEHOLD QUESTIONNAIRE [ ][ ]

LANGUAGE OF INTERVIEW [ ][ ]

01 FRENCH
02 TSHILUBA
03 LINGALA
04 OTHER LOCAL LANGUAGE ______________________

TRANSLATOR USED (YES = 1, NO = 2) [ ]

SUPERVISOR

NAME

NUMBER

INTRODUCTION AND CONSENT

Hello. My name is ____. I am working with POPULATION AND HEALTH RESEARCH INSTITUTE (PHERI) - WORLD HEALTH ORGANIZATION (WHO). We are conducting a survey about health among Kasaï displaced people in the Kasaï region and Kwilu province . The information we collect will help the government to plan health services. Your household was selected for the survey. I would like to ask you some questions about your household. The questions usually take about 15 to 20 minutes. All of the answers you give will be confidential and will not be shared with anyone other than members of our survey team. You don't have to be in the survey, but we hope you will agree to answer the questions since your views are important. If I ask you any question you don't want to answer, just let me know and I will go on to the next question or you can stop the interview at any time. In case you need more information about the survey, you may contact the person listed on this card.

IGNATURE OF INTERVIEWER ____________________ DATE ____________

RESPONDENT AGREES TO BE INTERVIEWED . . 1 ↓

RESPONDENT DOES NOT AGREE TO BE INTERVIEWED . . 2 → END

100	RECORD THE TIME.	HOURS . . . . [ ][ ]<br>MINUTE . . . . [ ][ ]

LINE NO.	USUAL RESIDENTS AND VISITORS	RELA<br>TO HI<br>HOUS
**101**	**102**	**1(**
	Please give me the names of the persons who usually live in your household and guests of the household who stayed here last night, starting with the head of the household.<br><br>AFTER LISTING THE NAMES AND RECORDING THE RELATIONSHIP AND SEX FOR EACH PERSON, ASK QUESTIONS 2A-2C TO BE SURE THAT THE LISTING IS COMPLETE.<br><br>THEN ASK APPROPRIATE QUESTIONS IN COLUMNS 5-20 FOR EACH PERSON.	What i<br>relatio<br>(NAM<br>head o<br>househ<br><br>SEE C<br>BELO

HOUSEHOLD CHARACTERISTICS

NO.	QUESTIONS AND FILTERS	CODING CATEGORIES	SKIP
201	What is the main source of drinking water for members of your household?	**PIPED WATER**<br>PIPED INTO DWELLING .... 11<br>PIPED TO YARD/PLC .... 12<br>PIPED TO NEIGHBOI .... 13<br>PUBLIC TAP/STANDPIP .... 14<br><br>TUBE WELL OR BOREHOL .... 21<br>**DUG WELL**<br>PROTECTED WE .... 31<br>UNPROTECTED WE .... 32<br>**WATER FROM SPRING**<br>PROTECTED SPRIN .... 41<br>UNPROTECTED SPRIN .... 42<br><br>RAINWATER .... 51<br>TANKER TRUCK .... 61<br>CART WITH SMALL TAI .... 71<br>SURFACE WATER (RIVER/DAM/<br>LAKE/POND/STREAM/CANAL/<br>IRRIGATION CHANNEL .... 81<br>BOTTLED WATEF .... 91<br><br>OTHER ____________ 96<br>(SPECIFY)	
202	What kind of toilet facility do members of your household usually use?<br><br>IF NOT POSSIBLE TO DETERMINE, ASK PERMISSION TO OBSERVE THE FACILITY.	**FLUSH OR POUR FLUSH TOILET**<br>FLUSH TO PIPED SEWER SYSTE .... 11<br>FLUSH TO SEPTIC TAN .... 12<br>FLUSH TO PIT LATRINE .... 13<br>FLUSH TO SOMEWHERE ELS .... 14<br>FLUSH, DON'T KNOW WHER .... 15<br>**PIT LATRINE**<br>VENTILATED IMPROVED PIT LATRI .... 21<br>PIT LATRINE WITH SLA .... 22<br>PIT LATRINE WITHOUT SLAB/OPEN .... 23<br><br>COMPOSTING TOILE .... 31<br>BUCKET TOILET .... 41<br>HANGING TOILET/HANGING LATRII .... 51<br>NO FACILITY/BUSH/FIELD .... 61<br><br>OTHER ____________ 96<br>(SPECIFY)	 <br><br><br><br><br><br><br><br><br><br><br><br>61 → 205
203	Do you share this toilet facility with other households?	YES .... 1<br>NO 2	 2 → 205
204	Including your own household, how many households use this toilet facility?	NO. OF HOUSEHOLDS<br>IF LESS THAN 10 .... [0][ ]<br><br>10 OR MORE HOUSEHOLD .... 95<br>DON'T KNOW 98	
205	What type of fuel does your household mainly use for cooking?	ELECTRICITY .... 01<br>LPG .... 02<br>NATURAL GAS .... 03<br>BIOGAS .... 04<br>KEROSENE .... 05<br>COAL, LIGNITE .... 06<br>CHARCOAL .... 07<br>WOOD .... 08<br>STRAW/SHRUBS/GRAS .... 09<br>AGRICULTURAL CROP .... 10<br>ANIMAL DUNG .... 11<br><br>NO FOOD COOKED IN HOUSEH .... 95<br><br>OTHER ____________ 96<br>(SPECIFY)	
206	How many rooms in this household are used for sleeping?	ROOMS .... [ ][ ]	
207	Does this household own any livestock, herds, other farm animals, or poultry?	YES .... 1<br>NO 2	 2 → 209
208	How many of the following animals does this household own?<br>IF NONE, RECORD '00'.<br>IF 95 OR MORE, RECORD '95'.<br>IF UNKNOWN, RECORD '98'.<br><br>a) Cows/ Bulls?<br>b) Other cattle?<br>c) Goats?<br>d) Sheep?<br>e) Chickens or other poultry?	YES NO<br><br>a) COWS/BULLS .... 1 2<br>b) OTHER CATTLE .... 1 2<br>c) GOATS .... 1 2<br>d) SHEEP .... 1 2<br>e) CHICKENS/POULTR 1 2	
209	Does your household have:<br><br>a) Electricity?<br>b) A radio?<br>c) A television?<br>d) A computer?<br>e) A refrigerator?	YES NO<br><br>a) ELECTRICITY .... 1 2<br>b) RADIO .... 1 2<br>c) TELEVISION .... 1 2<br>e) COMPUTER .... 1 2<br>f) REFRIGERATOR 1 2	
210	Does any member of this household own:<br><br>a) A watch?<br>b) A mobile phone?<br>c) A bicycle?<br>d) A motorcycle or motor scooter?<br>e) A car or truck?<br>f) A boat with a motor?	YES NO<br><br>a) WATCH .... 1 2<br>b) MOBILE PHON .... 1 2<br>c) BICYCLE .... 1 2<br>d) MOTORCYCLE/SCOOT .... 1 2<br>e) CAR/TRUCK .... 1 2<br>f) BOAT WITH 1 2	
211	Does any member of this household have a bank account?	YES .... 1<br>NO 2	

HOUSING CHARACTERISTCS

NO.	QUESTIONS AND FILTERS	CODING CATEGORIES		SKIP
212	MAIN MATERIAL OF THE FLOOR.<br><br>RECORD OBSERVATION.	**NATURAL FLOOR** (EARTH/SAND/DUNG)	11	
		**RUDIMENTARY FLOOR** (WOOD PLANKS/BAMBO)	21	
		**FINISHED FLOOR**		
		PARQUET OR POLISHED WOOD	31	
		VINYL OR ASPHALT STRIPS	32	
		CERAMIC TILES	33	
		CEMENT	34	
		OTHER ______ **(SPECIFY)**	96	
213	MAIN MATERIAL OF THE ROOF.<br><br>RECORD OBSERVATION.	**NATURAL ROOFING**		
		NO ROOF	11	
		THATCH/PALM LEAVES	12	
		SOD	13	
		**RUDIMENTARY ROOFING** (RUSTIC MAT/ BAMBOO/WOOD PLANKS	21	
		**FINISHED ROOFING**		
		ZINC/IRON SHEETS	31	
		CERAMIC TILES	34	
		CEMENT	35	
		OTHER ______ (SPECIFY)	96	
214	MAIN MATERIAL OF THE WALLS.<br><br>RECORD OBSERVATION.	**NATURAL WALLS**		
		NO WALLS	11	
		CANE/PALM/TRUNKS	12	
		DIRT	13	
		**RUDIMENTARY WALLS**		
		BAMBOO WITH MUD	21	
		STONE WITH MUD	22	
		UNCOVERED ADOBE	23	
		PLYWOOD	24	
		CARDBOARD	25	
		REUSED WOOD	26	
		**FINISHED WALLS**		
		CEMENT	31	
		STONE WITH LIME/CEMENT	32	
		BRICKS	33	
		CEMENT BLOCKS	34	
		COVERED ADOBE	35	
		WOOD PLANKS/SHINGLES	36	
		OTHER ______ (SPECIFY)	96	

INTERVIEWER'S OBSERVATIONS

TO BE FILLED IN AFTER COMPLETING INTERVIEW

COMMENTS ABOUT INTERVIEW:

COMMENTS ON SPECIFIC QUESTIONS:

ANY OTHER COMMENTS:

SUPERVISOR'S OBSERVATIONS
