# Supplementary material for: Situation analysis for delivering integrated comprehensive sexual and reproductive health services for displaced population of Kasaï, Democratic Republic of Congo: Protocol for a mixed method study
Source: PLoS One. 2020 Dec 21;15(12):e0242046. doi: 10.1371/journal.pone.0242046 (PMC7751877; doi:10.1371/journal.pone.0242046)
Supplement: S2 Annex — (DOCX) [file pone.0242046.s003.docx]

**Section 1: General Information about the respondent**

| NAME AND LINE NUMBER OF WOMAN |  |
| --- | --- |
| Participant ID: |  |
| Camp/ Village number |  |
| Name of Head of household |  |
| Household Number |  |
| Interview start time |  |
| Interview end time |  |
|  |  |

**Informed Consent**

Hello. My name is _______________________________________. I am working with POPULATION AND HEALTH RESEARCH INSTITUTE/ PROGRAMME NATIONAL DE LA SANTE DES ADOLESCENTS. We are conducting a survey about health and other topics all over [NAME OF PROVINCE]. The information we collect will help the government to plan health services. Your household was selected for the survey. The questions usually take about 30 to 60 minutes. All of the answers you give will be confidential and will not be shared with anyone other than members of our survey team. You don't have to be in the survey, but we hope you will agree to answer the questions since your views are important. If I ask you any question you don't want to answer, just let me know and I will go on to the next question or you can stop the interview at any time.

In case you need more information about the survey, you may contact the person listed on the card that has already been given to your household.

Do you have any questions?

May I begin the interview now?

SIGNATURE OF INTERVIEWER_______________________

Date _______________________

End the survey if the respondent did not agree to participate.

**Section 2: Socio-demographic characteristics**

| **No.** | **Questions and instructions** | **Coding categories** | | | | | **Skip** |
| --- | --- | --- | --- | --- | --- | --- | --- |
| 201 | When did you first arrive in this village/camp | Months \|__\|__\|  Years \|__\|__\| | | | | |  |
| 202 | How long have you been living continuously in (NAME OF CURRENT CAMP/VILLAGE OR CITY OF RESIDENCE)? | Months \|__\|__\|  Years \|__\|__\| | | | | |  |
| 203 | Just before you moved here, where have you been living (city/ village, Municipality and Province)? | Village/City________________________  Municipality________________________  Province___________________________ | | | | |  |
| 204 | In what month and year were you born? | Months \|__\|__\|  Years \|__\|__\| | | | | |  |
| 205 | How old were you at your last birthday? (*Instruction: If she could not tell her age, then if married - ask how many years she has been married, approximate age at marriage, approximate age during 1^st^ menstruation, age of 1^st^ child/approximate age at 1^st^ pregnancy, after how many years of marriage she got pregnant for the 1^st^ time. If unmarried- ask approximate age during 1^st^ menstruation, how many years having menstruation, years of schooling, age at enrollment in school, study gap in years*) | Years \|__\|__\| | | | | |  |
| 206 | What is your religion? | Catholic……………………………...1  Protestant ……………………………2  Pentecostal …………………………..3  Other Christian ………………………4  Others _______________________ 96  Specify | | | | |  |
| 207 | What is your marital status? *(If husband is currently living with her or not, or the husband got married to another place and live separately- these things need to be known by asking)* | Never married...……..………………………. 1  In union living with husband here……………….2  In union living not in this village ……………….3  Separate/ Divorce……. .………..…. ………… .4  Widow……………………………………... 5  Did not answer ………………….. 99 | | | | |  |
| 208 | Who is the sole decision maker in your family? [Probe: Makes the important decisions of the family]   - Marriage of children/education - Spending money - Any important decision in family |  | Yes | | No | |  |
|  |  | Myself | 1 | | 0 | |  |
|  |  | Husband | 1 | | 0 | |  |
|  |  | Father | 1 | | 0 | |  |
|  |  | Mother | 1 | | 0 | |  |
|  |  | Brother | 1 | | 0 | |  |
|  |  | Other Relative | 1 | | 0 | |  |
|  |  | Majhi | 1 | | 0 | |  |
|  |  | Others (Specify) …………….…….…………. 96 | | | | |  |
|  |  | Did not answer…………….…………………. 99 | | | | |  |
| 209 | Can you read? For example, a newspaper, book etc.? | Yes ………………………………..1  No …………………………………2  Did not answer ………………….. 99 | | | | | 213  213 |
| 210 | Have you ever-attended school? | Yes ………………………………..1  No …………………………………2  Did not answer ………………….. 99 | | | | | 213  213 |
| 211 | What is the highest level of education you completed? [Check with Ques 2.10 to be consistent, and correct Ques 2.10 if necessary]   - Have you completed the examination for the class you have studied highest? | Primary ……………………………1  Secondary …………………………2  University …………………………3  Post Graduate ……………………..4  Did not answer ………………….. 99 | | | | |  |
| 212 | What type of work you are engaged for earning money?  Please probe with each option mentioned  (Multiple Response) |  | | Yes | | No |  |
|  |  | Housekeeping (at other’s home/hotel) | | 1 | | 0 |  |
|  |  | Works at Field | | 1 | | 0 |  |
|  |  | Catches Fish | | 1 | | 0 |  |
|  |  | Fuel-wood collection | | 1 | | 0 |  |
|  |  | Construction site labor | | 1 | | 0 |  |
|  |  | Dily Wage Labor (other than 1, 2 and 3) | | 1 | | 0 |  |
|  |  | Own small business  (e.g. grocery, selling vegetables, etc.) | | 1 | | 0 |  |
|  |  | Monthly-wage labor  (e.g. community health worker, service at health center/WFC/NGO office, etc.) | | 1 | | 0 |  |
|  |  | Fishing | | 1 | | 0 |  |
|  |  | Currently not working | | 1 | | 0 |  |
|  |  | Others (Specify) …………….………………. 96 | | | | |  |
|  |  | Did not answer…………….……………...…. 99 | | | | |  |
| 213 | What is the main source of your family income?  [Multiple responses possible] |  | | Yes | | No |  |
|  |  | Respondent’s income | | 1 | | 0 |  |
|  |  | Relief | | 1 | | 0 |  |
|  |  | Fuel-wood collection | | 1 | | 0 |  |
|  |  | Daily wage labor | | 1 | | 0 |  |
|  |  | Own small business  (e.g. grocery, selling vegetables, etc.) | | 1 | | 0 |  |
|  |  | Monthly-wage labor  (e.g., teacher, other service, etc.) | | 1 | | 0 |  |
|  |  | Others (Specify) …………….………………. 96 | | | | |  |
|  |  | Did not answer…………….…...……………. 99 | | | | |  |
| 214 | What is your family’s monthly expenditure in last month?  *Probe: (ask for approximate amount for*   - *Food* - *Clothes* - *Education* - *Other expenses*) | Congo Francs \|__\|__\|__\|__\|__\|  US Dollars. \|__\|__\|__\|__\|__\| | | | | |  |

**Type of Participant:** *(Surveyor will fill this up according to the age of the respondent mentioned in the question 2.3)*

- Women
- Adolescent

*Based on the category next section of questions will be appeared.*

**Section 3: Menstrual health (only for adolescent girls aged 12-18)**

| **No.** | **Questions and instructions** | **Coding categories** | | | **Skip** |
| --- | --- | --- | --- | --- | --- |
| 301 | Has your menstruation started? | Yes …………………………………1  No ……………………………….…2  Did not answer……………………. 99 | | | 503  501 |
| 302 | How many months/years back, did you have your first menstruation? | Number of Months \|__\|__\| | | |  |
| 303 | What do you usually use during menstruation?  (Multiple Response) |  | Yes | No |  |
|  |  | Cloth | 1 | 0 |  |
|  |  | Cotton | 1 | 0 |  |
|  |  | Sanitary | 1 | 0 |  |
|  |  | Toilet Paper | 1 | 0 |  |
|  |  | Others (Specify) …………….………………. 96 | | |  |
| 304 | Do you have any of the symptoms when you have periods?  MULTIPLE RESPONSE |  | Yes | No | 308 |
|  |  | Abdominal pain | 1 | 0 |  |
|  |  | Lower back pain | 1 | 0 |  |
|  |  | Pain in upper leg and thighs | 1 | 0 |  |
|  |  | Nausea | 1 | 0 |  |
|  |  | Tiredness | 1 | 0 |  |
|  |  | Body-aches | 1 | 0 |  |
|  |  | Dizziness | 1 | 0 |  |
|  |  | Headaches | 1 | 0 |  |
|  |  | Joint pains | 1 | 0 |  |
|  |  | Tender breasts | 1 | 0 |  |
|  |  | No symptoms | 1 | 0 |  |
|  |  | Others (Specify)…………….………………. 96 | | |  |
|  |  | Did not answer……………………………… 99 | | |  |
| 305 | What do you do when you have any of those symptoms?  MULTIPLE RESPONSE |  | Yes | No |  |
|  |  | Take rest | 1 | 0 |  |
|  |  | Drink warm water | 1 | 0 |  |
|  |  | Take hot shower/ hot biting | 1 | 0 |  |
|  |  | Take medicine from local drug-store | 1 | 0 |  |
|  |  | No measures taken | 1 | 0 |  |
|  |  | Others (Specify)…………….………………. 96 | | |  |
|  |  | Did not answer……………………………… 99 | | |  |
| 306 | Have you ever consulted with anyone about the symptoms you have during menstruation? |  | Yes | No | 3.8      308 |
|  |  | Didn’t consult with anyone | 1 | 0 |  |
|  |  | Mother/sister/family member | 1 | 0 |  |
|  |  | Husband | 1 | 0 |  |
|  |  | Relative | 1 | 0 |  |
|  |  | Friends | 1 | 0 |  |
|  |  | Neighbor | 1 | 0 |  |
|  |  | Health Worker visited home | 1 | 0 |  |
|  |  | Doctor/Nurse/Paramedic | 1 | 0 |  |
|  |  | Local Traditional healer | 1 | 0 |  |
|  |  | Others (Specify)…………….………………. 96 | | |  |
|  |  | Did not answer……………………………… 99 | | |  |
| 307 | Where did you go to consult with doctor/nurse/paramedic?  (Instruction: If the respondent says any name, probe if that is healthcare facility) | Healthcare Facility (Specify) ……...1  Others (Specify)…………………. 96  Didn’t answer …………………….99 | | |  |
| 308 | Do you have restriction to any certain behavior during your period?  (Multiple Response) |  | Yes | No |  |
|  |  | Restricted mobility outside home | 1 | 0 |  |
|  |  | Certain places are restricted to go  (e.g. kitchen, graveyard, fetching water/ponds, etc.) | 1 | 0 |  |
|  |  | Certain foods are restricted  (e.g. certain type of fish, meat, sour etc.) | 1 | 0 |  |
|  |  | Separate sleeping area | 1 | 0 |  |
|  |  | Not allowed to sweep the house | 1 | 0 |  |
|  |  | Not allowed to keep the hair untied | 1 | 0 |  |
|  |  | No restrictions | 1 | 0 |  |
|  |  | Others (Specify) ………………………….96 | | |  |
|  |  | Didn’t answer …………………………… 99 | | |  |

**Section 4: Pregnancy and Delivery care**

| **No.** | **Questions and instructions** | **Coding categories** | | | | | **Skip** |
| --- | --- | --- | --- | --- | --- | --- | --- |
| 401 | Have you ever been pregnant | Yes ………………………………...1  No …………………………………2  Did not answer……………….…...99 | | | | | 501  501 |
| 402 | Total number of pregnancies? | \|__\|__\| | | | | |  |
| 403 | Have you ever given birth? | Yes ……………………………….1  No …………………………………2  Didn’t answer …………………….99 | | | | | Next section |
| 403 | How many of your son live with you at your house currently? | Number of sons \|__\|__\| | | | | |  |
| 404 | How many of your daughter live with you at your house currently? | Number of daughters \|__\|__\| | | | | |  |
| 404 | How many son live separated from you? | Sons live elsewhere \|__\|__\| | | | | |  |
| 404 | How many daughters live separated from you? | Daughters live elsewhere \|__\|__\| | | | | |  |
| 405 | How many of your boy child was born alive but died? *[If none, put 00 in two boxes]* | Boys dead \|__\|__\| | | | | |  |
| 406 | How many of your girl child was born alive but died?  *[If none, put 00 in two boxes]* | Girls dead \|__\|__\| | | | | |  |
| 407 | How many miscarriage or Menstrual Regulation you had?  *[If none, put 00 in two boxes]* | Number of pregnancies terminated \|__\| | | | | |  |
| 408 | How many still births did you had? | Number of dead child/children \|__\| | | | | |  |
|  | **Now I would like to ask you about your pregnancy history after coming here (this village/ camp)** | | | | | | |
| 409 | Are you pregnant now? | Yes ………………………………...1  No …………………………………2  Did not answer……………….…...99 | | | | | 422  422 |
| 410 | For How many months in your pregnancy are you at? | Number of months \|__\|__\| | | | | |  |
| 411 | Are you following up with anyone for antenatal care for this pregnancy? | Yes ………………………………...1  No …………………………………2  Didn’t answer …………………….99 | | | | | 417  418 |
| 412 | Who are you following up with?  Probe to identify each type  [Multiple response possible] |  | | Yes | | No |  |
|  |  | Health worker visited home | | 1 | | 0 |  |
|  |  | Local TBA at home | | 1 | | 0 |  |
|  |  | Doctor/Nurse/Midwife/Paramedic at a health facility/clinic | | 1 | | 0 |  |
|  |  | Burmese /Private Doctor at a health facility/clinic | | 1 | | 0 |  |
|  |  | Drug-shop/Pharmacy | | 1 | | 0 |  |
|  |  | Others Specify ………………….96 | | | | |  |
|  |  | Didn’t answer ………………….99 | | | | |  |
| 413 | Where did you receive antenatal care for this pregnancy?  (Instruction: If the respondent says any name, probe if that is healthcare facility) | Home ………………………………1  Healthcare Facility (Specify) ……...2  Others (Specify)…………………. 96  Didn’t answer …………………….99 | | | | |  |
| 414 | How many antenatal care visits did you receive during this pregnancy? | Never ……………………………. 1  Once ……………………………. 2  Twice …………………………… 3  Four Times ……………………… 4  Others (Specify)……………....... 96  Don't know . . . . . . . . . ………….98  Didn’t answer ………………...... 99 | | | | | 420 |
| 415 | As part of your antenatal care during this pregnancy, were any of the following done at least once?   - Was your weight measured? - Was your blood pressure measured? - Did you have a urine test? - Did you have a blood test? - Did you have an ultrasonography? - Were you counselled about danger signs? - Was your fundus height checked? (Did they use any machine to check your abdomen?) |  | | Yes | | No | 421 |
|  |  | Weight | | 1 | | 0 |  |
|  |  | Height | | 1 | | 0 |  |
|  |  | Blood Pressure | | 1 | | 0 |  |
|  |  | Urine | | 1 | | 0 |  |
|  |  | Bloodtest | | 1 | | 0 |  |
|  |  | Ultrasound | | 1 | | 0 |  |
|  |  | Advice Danger Sign | | 1 | | 0 |  |
|  |  | Abdominal Test | | 1 | | 0 |  |
|  |  | Titanus (TT) | | 1 | | 0 |  |
|  |  | Others | | 1 | | 0 |  |
|  |  | Don’t Know …………………….98 | | | | |  |
|  |  | Didn’t Answer ………………….99 | | | | |  |
| 416 | (If you didn’t receive antenatal care), What was the reason for not receiving antenatal care during this pregnancy? |  | | Yes | | No |  |
|  |  | Did not feel the necessity | | 1 | | 0 |  |
|  |  | Did not know where/who to go | | 1 | | 0 |  |
|  |  | Did not know such care | | 1 | | 0 |  |
|  |  | Health center is far/Access to facility was/is difficult | | 1 | | 0 |  |
|  |  | Services were/are not available here | | 1 | | 0 |  |
|  |  | Did not like services provided in the facilities | | 1 | | 0 |  |
|  |  | Family/Husband did not allow | | 1 | | 0 |  |
|  |  | Did not have money | | 1 | | 0 |  |
|  |  | Nobody to accompany | | 1 | | 0 |  |
|  |  | Previous experience is not good | | 1 | | 0 |  |
|  |  | Concerned about being treated by a male doctor | | 1 | | 0 |  |
|  |  | Afraid to go health care center | | 1 | | 0 |  |
|  |  | Hadn’t time | | 1 | | 0 |  |
|  |  | Neighbor forbade to go | | 1 | | 0 |  |
|  |  | Health care center was far away from home | | 1 | | 0 |  |
|  |  | Others (Specify) …………………96 | | | | |  |
|  |  | Didn’t Answer ………………….99 | | | | |  |
| 417 | During this pregnancy, do /did you have any pregnancy related problems or complications? (after arriving in this camp)  MULTIPLE RESPONSE |  | | Yes | | No | 424 |
|  |  | Bleeding | | 1 | | 0 |  |
|  |  | Fever | | 1 | | 0 |  |
|  |  | Frequent Vomiting | | 1 | | 0 |  |
|  |  | Headache/ Blurred Vision | | 1 | | 0 |  |
|  |  | Edema | | 1 | | 0 |  |
|  |  | Convulsion | | 1 | | 0 |  |
|  |  | Abdominal Pain | | 1 | | 0 |  |
|  |  | Less/no movement of the Fetus | | 1 | | 0 |  |
|  |  | No Problem | | 1 | | 0 |  |
|  |  | Others (Specify) …………………….96 | | | | |  |
|  |  | Didn’t Answer ………………………99 | | | | |  |
| 418 | Did you seek help for the problem(s) or complication(s)? | Yes ……………………………….1  No …………………………………2  Didn’t answer ……………… 99 | | | | | 423  424 |
| 419 | Where did you seek help?  (Instruction: If the respondent says any name, probe if that is healthcare facility) | Home ………………………………1  Healthcare Facility (Specify) ……...2  Others (Specify)…………………. 96  Didn’t answer …………………….99 | | | | | 424 |
| 420 | (If you didn’t seek service), What was the reason for not seeking help / service for the complication / problem you had during you’re this pregnancy?  (Multiple Response) |  | | Yes | | No |  |
|  |  | Did not feel the necessity | | 1 | | 0 |  |
|  |  | Did not know where/who to go | | 1 | | 0 |  |
|  |  | Did not know such care | | 1 | | 0 |  |
|  |  | Health center is far/Access to facility was/is difficult | | 1 | | 0 |  |
|  |  | Services were/are not available here | | 1 | | 0 |  |
|  |  | Did not like services provided in the facilities | | 1 | | 0 |  |
|  |  | Family/Husband did not allow | | 1 | | 0 |  |
|  |  | Did not have money | | 1 | | 0 |  |
|  |  | Nobody to accompany | | 1 | | 0 |  |
|  |  | Previous experience is not good | | 1 | | 0 |  |
|  |  | Concerned about being treated by a male doctor | | 1 | | 0 |  |
|  |  | Afraid to go health care center | | 1 | | 0 |  |
|  |  | Hadn’t time | | 1 | | 0 |  |
|  |  | Neighbor forbade to go | | 1 | | 0 |  |
|  |  | Health care center was far away from home | | 1 | | 0 |  |
|  |  | Others (Specify) …………………….96 | | | | |  |
|  |  | Didn’t Answer ………………………99 | | | | |  |
| 421 | Were you pregnant after coming to this camp?  Instruction: The pregnancy before current pregnancy | Yes ………………………………...1  No …………………………………2 | | | | | 435 |
| 422 | Did you see anyone for antenatal care for last pregnancy? | Yes ………………………………...1  No …………………………………2  Didn’t answer …………………….99 | | | | | 430 |
| 423 | Whom did you see?  Probe to identify each type  [Multiple response possible] |  | | Yes | | No |  |
|  |  | Health worker visited home | | 1 | | 0 |  |
|  |  | Local TBA at home | | 1 | | 0 |  |
|  |  | Doctor/Nurse/Midwife/Paramedic at a health facility/clinic | | 1 | | 0 |  |
|  |  | Burmese /Private Doctor at a health facility/clinic | | 1 | | 0 |  |
|  |  | Drug-shop/Pharmacy | | 1 | | 0 |  |
|  |  | Others Specify ………………….96 | | | | |  |
|  |  | Didn’t answer ………………….99 | | | | |  |
| 424 | Where did you receive antenatal care for last pregnancy?  (Instruction: If the respondent says any name, probe if that is healthcare facility) | Home ………………………………1  Healthcare Facility (Specify) ……...2  Others (Specify)…………………. 96  Didn’t answer …………………….99 | | | | |  |
| 425 | How many times did you receive antenatal care during last pregnancy? | Never ……………………………. 1  Once ……………………………. 2  Twice …………………………… 3  Four Times ……………………… 4  Others (Specify)……………....... 96  Don't know . . . . . . . . . ………….98  Didn’t answer ………………...... 99 | | | | | 430 |
| 426 | As part of your antenatal care during last pregnancy, were any of the following done at least once?   - Was your weight measured? - Was your blood pressure measured? - Did you have a urine test? - Did you have a blood test? - Did you have an ultrasonography? - Were you counselled about danger signs?   Was your fundus height checked? (Did they use any machine to check your abdomen?) |  | Yes | | No | | 431 |
|  |  | Weight | 1 | | 0 | |  |
|  |  | Height | 1 | | 0 | |  |
|  |  | Blood Pressure | 1 | | 0 | |  |
|  |  | Urine | 1 | | 0 | |  |
|  |  | Blood test | 1 | | 0 | |  |
|  |  | Ultrasound | 1 | | 0 | |  |
|  |  | Advice Danger Sign | 1 | | 0 | |  |
|  |  | Abdominal Test | 1 | | 0 | |  |
|  |  | Tetanus (TT) | 1 | | 0 | |  |
|  |  | Others | 1 | | 0 | |  |
|  |  | Don’t Know ……………………..98 | | | | |  |
|  |  | Didn’t Answer …………………..99 | | | | |  |
| 427 | (If you didn’t receive antenatal care), What was the reason for not receiving antenatal care during your last pregnancy? |  | Yes | | No | |  |
|  |  | Did not feel the necessity | 1 | | 0 | |  |
|  |  | Did not know where/who to go | 1 | | 0 | |  |
|  |  | Did not know such care | 1 | | 0 | |  |
|  |  | Health center is far/Access to facility was/is difficult | 1 | | 0 | |  |
|  |  | Services were/are not available here | 1 | | 0 | |  |
|  |  | Did not like services provided in the facilities | 1 | | 0 | |  |
|  |  | Family/Husband did not allow | 1 | | 0 | |  |
|  |  | Did not have money | 1 | | 0 | |  |
|  |  | Nobody to accompany | 1 | | 0 | |  |
|  |  | Previous experience is not good | 1 | | 0 | |  |
|  |  | Concerned about being treated by a male doctor | 1 | | 0 | |  |
|  |  | Afraid to go health care center | 1 | | 0 | |  |
|  |  | Hadn’t time | 1 | | 0 | |  |
|  |  | Neighbor forbade to go | 1 | | 0 | |  |
|  |  | Health care center was far away from home | 1 | | 0 | |  |
|  |  | Others (Specify) …………………96 | | | | |  |
|  |  | Didn’t Answer ………………….99 | | | | |  |
| 428 | During last pregnancy, what type of problems or complications you had?  MULTIPLE RESPONSE |  | Yes | | No | | 435 |
|  |  | Bleeding | 1 | | 0 | |  |
|  |  | Fever | 1 | | 0 | |  |
|  |  | Frequent Vomiting | 1 | | 0 | |  |
|  |  | Headache/ Blurred Vision | 1 | | 0 | |  |
|  |  | Edema | 1 | | 0 | |  |
|  |  | Convulsion | 1 | | 0 | |  |
|  |  | Abdominal Pain | 1 | | 0 | |  |
|  |  | Less/no movement of the Fetus | 1 | | 0 | |  |
|  |  | No Problem | 1 | | 0 | |  |
|  |  | Others (Specify) …………………….96 | | | | |  |
|  |  | Didn’t Answer ………………………99 | | | | |  |
| 429 | Did you seek help for the problem(s) or complication(s)? | Yes ……………………………….1  No …………………………………2  Didn’t answer ……………… 99 | | | | | 434 |
| 430 | Where did you seek help?  (Instruction: If the respondent says any name, probe if that is healthcare facility) | Home ………………………………1  Healthcare Facility (Specify) ……...2  Others (Specify)…………………. 96  Didn’t answer …………………….99 | | | | |  |
| 431 | (If you didn’t seek service), What was the reason for not seeking help / service for the complication / problem you had during your last pregnancy?  (Multiple Response) |  | Yes | | No | |  |
|  |  | Did not feel the necessity | 1 | | 0 | |  |
|  |  | Did not know where/who to go | 1 | | 0 | |  |
|  |  | Did not know such care | 1 | | 0 | |  |
|  |  | Health center is far/Access to facility was/is difficult | 1 | | 0 | |  |
|  |  | Services were/are not available here | 1 | | 0 | |  |
|  |  | Did not like services provided in the facilities | 1 | | 0 | |  |
|  |  | Family/Husband did not allow | 1 | | 0 | |  |
|  |  | Did not have money | 1 | | 0 | |  |
|  |  | Nobody to accompany | 1 | | 0 | |  |
|  |  | Previous experience is not good | 1 | | 0 | |  |
|  |  | Concerned about being treated by a male doctor | 1 | | 0 | |  |
|  |  | Afraid to go health care center | 1 | | 0 | |  |
|  |  | Hadn’t time | 1 | | 0 | |  |
|  |  | Neighbor forbade to go | 1 | | 0 | |  |
|  |  | Health care center was far away from home | 1 | | 0 | |  |
|  |  | Others (Specify) …………………….96 | | | | |  |
|  |  | Didn’t Answer ………………………99 | | | | |  |
| 432 | Did you give birth to a child after coming here? | Yes ……………………………….1  No …………………………………2  Didn’t answer …………………….99 | | | | | Next section |
| 433 | When did you delivered a child last time?  (Less than 1 month and 15 days will be 1 month) | Months ago \|__\|__\| | | | | |  |
| 434 | What was the mode of delivery? | Normal vaginal …………………….1  Caesarian …………………………...2 | | | | |  |
| 435 | Is the baby alive? | Yes ……………………………….1  No …………………………………2  Didn’t answer …………………….99 | | | | |  |
| 436 | Where did you give birth?  (Instruction: If the respondent says any name, probe if that is healthcare facility) | Home ………………………………1  Healthcare Facility (Specify) ……...2  Others (Specify)…………………. 96  Didn’t answer …………………….99 | | | | | 442 |
| 437 | Who helped you during your child birth?  (Multiple Question) |  | | Yes | | No |  |
|  |  | Family member/relative/neighbor | | 1 | | 0 |  |
|  |  | Health worker who visited the home | | 1 | | 0 |  |
|  |  | Local traditional healer | | 1 | | 0 |  |
|  |  | Local drug store / Pharmacy | | 1 | | 0 |  |
|  |  | Local TBA | | 1 | | 0 |  |
|  |  | Others (Specify)…………………. .96 | | | | |  |
|  |  | Didn’t answer …………………….99 | | | | |  |
| 438 | (**If you had home delivery**), What was the reason for not going to any health facility for delivering your child?  SKIP IF DELIVERED AT ANY HEALTH FACILITY |  | | Yes | | No |  |
|  |  | Did not feel the necessity | | 1 | | 0 |  |
|  |  | Did not know where/who to go | | 1 | | 0 |  |
|  |  | Did not know such care | | 1 | | 0 |  |
|  |  | Health center is far/Access to facility was/is difficult | | 1 | | 0 |  |
|  |  | Services were/are not available here | | 1 | | 0 |  |
|  |  | Did not like services provided in the facilities | | 1 | | 0 |  |
|  |  | Family/Husband did not allow | | 1 | | 0 |  |
|  |  | Did not have money | | 1 | | 0 |  |
|  |  | Nobody to accompany | | 1 | | 0 |  |
|  |  | Previous experience is not good | | 1 | | 0 |  |
|  |  | Concerned about being treated by a male doctor | | 1 | | 0 |  |
|  |  | Afraid to go health care center | | 1 | | 0 |  |
|  |  | Hadn’t time | | 1 | | 0 |  |
|  |  | Neighbor forbade to go | | 1 | | 0 |  |
|  |  | Health care center was far away from home | | 1 | | 0 |  |
|  |  | Others (Specify) …………………….96 | | | | |  |
|  |  | Didn’t Answer ………………………99 | | | | |  |
| 439 | During last child birth, did you have any complication? | Yes …………………………………… 1  No ……………………………………. 2  Didn’t answer ………………………… 99 | | | | | 446 |
| 440 | What type of problem(s) or complication(s) did you have?  MULTIPLE RESPONSE |  | | Yes | | No |  |
|  |  | Heavy bleeding | | 1 | | 0 |  |
|  |  | Prolong labor | | 1 | | 0 |  |
|  |  | Obstructed labor | | 1 | | 0 |  |
|  |  | Fever | | 1 | | 0 |  |
|  |  | Edema | | 1 | | 0 |  |
|  |  | Convulsion | | 1 | | 0 |  |
|  |  | Retained Placenta | | 1 | | 0 |  |
|  |  | Others (Specify)…………………. 96 | | | | |  |
|  |  | Do not know……………………... 98 | | | | |  |
|  |  | Didn’t answer …………………….99 | | | | |  |
| 441 | Where did you seek help for the problem(s) or complication(s)?  (Instruction: If the respondent says any name, probe if that is healthcare facility) | Did not seek any help ……………. 1  Home ………………………………2  Healthcare Facility (Specify) ……...3  Others (Specify)…………………. 96  Didn’t answer …………………….99 | | | | | 446 |
| 442 | (If you didn’t seek service), What was the reason for not seeking help / service for the complication / problem you had during delivery?  MULTIPLE RESPONSE |  | | Yes | | No |  |
|  |  | Did not feel the necessity | | 1 | | 0 |  |
|  |  | Did not know where/who to go | | 1 | | 0 |  |
|  |  | Did not know such care | | 1 | | 0 |  |
|  |  | Health center is far/Access to facility was/is difficult | | 1 | | 0 |  |
|  |  | Services were/are not available here | | 1 | | 0 |  |
|  |  | Did not like services provided in the facilities | | 1 | | 0 |  |
|  |  | Family/Husband did not allow | | 1 | | 0 |  |
|  |  | Did not have money | | 1 | | 0 |  |
|  |  | Nobody to accompany | | 1 | | 0 |  |
|  |  | Previous experience is not good | | 1 | | 0 |  |
|  |  | Concerned about being treated by a male doctor | | 1 | | 0 |  |
|  |  | Afraid to go health care center | | 1 | | 0 |  |
|  |  | Hadn’t time | | 1 | | 0 |  |
|  |  | Neighbor forbade to go | | 1 | | 0 |  |
|  |  | Health care center was far away from home | | 1 | | 0 |  |
|  |  | Others (Specify) …………………….96 | | | | |  |
|  |  | Didn’t Answer ………………………99 | | | | |  |
| 443 | Did you see anyone for postnatal care after delivery? | Yes ……………………………….1  No …………………………………2  Didn’t answer …………………….99 | | | | | 450  Next section |
| 444 | Whom did you see? |  | | Yes | | No |  |
|  |  | Health worker visited home | | 1 | | 0 |  |
|  |  | Local TBA | | 1 | | 0 |  |
|  |  | Doctor/Nurse/ Midwife/paramedic | | 1 | | 0 |  |
|  |  | Burma Doctor/Privet doctor | | 1 | | 0 |  |
|  |  | Drug Store | | 1 | | 0 |  |
|  |  | Others (Specify)……………………. 96 | | | | |  |
|  |  | Didn’t answer ……………………….99 | | | | |  |
| 445 | Where did you receive postnatal care for this /last pregnancy?  (Instruction: If the respondent says any name, probe if that is healthcare facility) | Home ………………………………1  Healthcare Facility (Specify) ……...2  Others (Specify)…………………. 96  Didn’t answer …………………….99 | | | | |  |
| 446 | How many times did you receive postnatal  care after delivery? | Never……………………………….1  Once……………………………......2  Twice……………………………….3  Thrice………………………….…...4  Four Time……………………….5  Others………………………….96  Don't know ……………………98  Didn't answer………………….99 | | | | | Next  Section |
| 447 | (If you didn’t receive postnatal care), What was the reason for not receiving postnatal care after delivery?  MULTIPLE RESPONSE |  | | Yes | | No |  |
|  |  | Did not feel the necessity | | 1 | | 0 |  |
|  |  | Did not know where/who to go | | 1 | | 0 |  |
|  |  | Did not know such care | | 1 | | 0 |  |
|  |  | Health center is far/Access to facility was/is difficult | | 1 | | 0 |  |
|  |  | Services were/are not available here | | 1 | | 0 |  |
|  |  | Did not like services provided in the facilities | | 1 | | 0 |  |
|  |  | Family/Husband did not allow | | 1 | | 0 |  |
|  |  | Did not have money | | 1 | | 0 |  |
|  |  | Nobody to accompany | | 1 | | 0 |  |
|  |  | Previous experience is not good | | 1 | | 0 |  |
|  |  | Concerned about being treated by a male doctor | | 1 | | 0 |  |
|  |  | Afraid to go health care center | | 1 | | 0 |  |
|  |  | Hadn’t time | | 1 | | 0 |  |
|  |  | Neighbor forbade to go | | 1 | | 0 |  |
|  |  | Health care center was far away from home | | 1 | | 0 |  |
|  |  | Others (Specify) …………………….96 | | | | |  |
|  |  | Didn’t Answer ………………………99 | | | | |  |

**Section 5: Family Planning services**

| **No.** | **Questions and instructions** | **Coding categories** | | | | **Skip** |
| --- | --- | --- | --- | --- | --- | --- |
| 501 | Have you or your husband ever used anything to delay or prevent you from getting pregnant? | Yes ……………………………….1  No …………………………………2  Never had physical relation ……….3 | | | | 503  503 |
| 502 | What is/are the method(s) have you ever used?  CIRCLE ALL MENTIONED (MULTIPLE RESPONSE) |  | Yes | | No |  |
|  |  | Pill | 1 | | 0 |  |
|  |  | Injection | 1 | | 0 |  |
|  |  | Condom | 1 | | 0 |  |
|  |  | IUD | 1 | | 0 |  |
|  |  | Norplant/Implant | 1 | | 0 |  |
|  |  | Male sterilization | 1 | | 0 |  |
|  |  | Female sterilization | 1 | | 0 |  |
|  |  | Emergency Contraception Pill | 1 | | 0 |  |
|  |  | Safe period | 1 | | 0 |  |
|  |  | Withdrawal | 1 | | 0 |  |
|  |  | Others (Specify)…………………. 96 | | | |  |
|  |  | Didn’t answer …………………….99 | | | |  |
| 503 | What is your preferred method? |  | Yes | | No |  |
|  |  | Pill | 1 | | 0 |  |
|  |  | Injection | 1 | | 0 |  |
|  |  | Condom | 1 | | 0 |  |
|  |  | IUD | 1 | | 0 |  |
|  |  | Norplant/Implant | 1 | | 0 |  |
|  |  | Male sterilization | 1 | | 0 |  |
|  |  | Female sterilization | 1 | | 0 |  |
|  |  | Emergency Contraception Pill | 1 | | 0 |  |
|  |  | Safe period | 1 | | 0 |  |
|  |  | Withdrawal | 1 | | 0 |  |
|  |  | Others (Specify)…………………. 96 | | | |  |
|  |  | Didn’t answer …………………….99 | | | |  |
| 504 | Why do you prefer this method? | Easy to use ………………………. 1  Give me long time protection ……. 2  Don’t need husband’s permission .. 3  Adjust with my body …………….. 4  Using this since long …………….. 5  Others (Specify)…………………. 96  Didn’t answer …………………….99 | | | |  |
| 505 | What is/are the method(s) have you heard about?  (MULTIPLE RESPONSE) |  | Yes | | No | Next section |
|  |  | Pill | 1 | | 0 |  |
|  |  | Injection | 1 | | 0 |  |
|  |  | Condom | 1 | | 0 |  |
|  |  | IUD | 1 | | 0 |  |
|  |  | Norplant/Implant | 1 | | 0 |  |
|  |  | Male sterilization | 1 | | 0 |  |
|  |  | Female sterilization | 1 | | 0 |  |
|  |  | Emergency Contraception Pill | 1 | | 0 |  |
|  |  | Safe period | 1 | | 0 |  |
|  |  | Withdrawal | 1 | | 0 |  |
|  |  | \| Didn't hear anything \| \| --- \| | 1 | | 0 |  |
|  |  | Others (Specify) …………………. 96 | | | |  |
|  |  | Didn’t answer…………………….99 | | | |  |
| 506 | From where/whom this method can be obtained?  (Instruction: If the respondent says any name, probe if that is healthcare facility) | Home ………………………………1  Healthcare Facility (Specify) ……...2  Others (Specify)…………………. 96  Didn’t answer …………………….99 | | | |  |
| 507 | From where/whom have you heard about the names of these methods? |  | Yes | No | |  |
|  |  | Health worker visited home in here | 1 | 0 | |  |
|  |  | Doctor/Nurse from Barma | 1 | 0 | |  |
|  |  | Doctor/ Nurse in here | 1 | 0 | |  |
|  |  | Family Member (e.g. sister/mother/sister-in-law etc.) | 1 | 0 | |  |
|  |  | Neighbor | 1 | 0 | |  |
|  |  | Women Friendly Center | 1 | 0 | |  |
|  |  | Teacher | 1 | 0 | |  |
|  |  | Heard from others | 1 | 0 | |  |
|  |  | Others (Specify)…………………. 96 | | | |  |
|  |  | Didn’t answer …………………….99 | | | |  |
| 508 | When was the last time you used this (name of the method) method? | When I was in my country……….…1  Currently Using …….……….…. 2  1-2 months ago ……………….…3  3-6 months ago.……………….…4  more than 6 months ago.………...5  Not applicable………………….98  Didn’t answer …………………….99 | | | | Next  section |
| 509 | Why did you stop using this (name of the method) method?  Multiple Response |  | Yes | | No |  |
|  |  | Don’t know where to get | 1 | | 0 |  |
|  |  | Had physical problem | 1 | | 0 |  |
|  |  | Husband didn’t allow any more | 1 | | 0 |  |
|  |  | Wanted to conceive child | 1 | | 0 |  |
|  |  | Others (Specify)………………………96 | | | |  |

**Section 6: Menstrual Regulation (MR) and abortion**

| **No.** | **Questions and instructions** | **Coding categories** | | | **Skip** |
| --- | --- | --- | --- | --- | --- |
| **601** | Have you ever had a pregnancy that ended using menstrual regulation or was aborted after you came here? | Yes ……………………………….1  No …………………………………2  Didn’t answer …………………….99 | | | Next section |
| **602** | How did you do the Menstrual Regulation?  Multiple Question |  | Yes | No |  |
|  |  | Through Medicine | 1 | 0 |  |
|  |  | Went to Doctor | 1 | 0 |  |
|  |  | Went to traditional healer | 1 | 0 |  |
|  |  | Through TBA | 1 | 0 |  |
|  |  | Through machine | 1 | 0 |  |
|  |  | Others (Specify).…………………. 96 | | |  |
| **603** | How many times did you successfully terminate a pregnancy? | Number of times \|__\|__\| | | |  |
| **604** | When was the last time you terminated pregnancy? [*If they answer in months then multiply the number of months by 30*] | months \|__\|__\| | | |  |
| **605** | Did you seek any help for such pregnancy termination in the last time? | Yes ……………………………….1  No …………………………………2  Didn’t answer …………………….99 | | | 607 |
| **606** | From where you received help/services?  (Instruction: If the respondent says any name, probe if that is healthcare facility) | Home ………………………………1  Healthcare Facility (Specify) ……...2  Others (Specify)…………………. 96  Didn’t answer …………………….99 | | | 608 |
| **607** | (If you didn’t seek service), What was the reason for not seeking help / service?  Multiple Response |  | Yes | No |  |
|  |  | Did not feel the necessity | 1 | 0 |  |
|  |  | Did not know where/who to go | 1 | 0 |  |
|  |  | Did not know such care | 1 | 0 |  |
|  |  | Health center is far/Access to facility was/is difficult | 1 | 0 |  |
|  |  | Services were/are not available here | 1 | 0 |  |
|  |  | Did not like services provided in the facilities | 1 | 0 |  |
|  |  | Family/Husband did not allow | 1 | 0 |  |
|  |  | Did not have money | 1 | 0 |  |
|  |  | Nobody to accompany | 1 | 0 |  |
|  |  | Previous experience is not good | 1 | 0 |  |
|  |  | Concerned about being treated by a male doctor | 1 | 0 |  |
|  |  | Afraid to go health care center | 1 | 0 |  |
|  |  | Hadn’t time | 1 | 0 |  |
|  |  | Neighbor forbade to go | 1 | 0 |  |
|  |  | Health care center was far away from home | 1 | 0 |  |
|  |  | Others (Specify) …………………….96 | | |  |
|  |  | Didn’t Answer ………………………99 | | |  |
| **608** | Was there any sort of complication related to your last pregnancy termination? | Yes ……………………………….1  No …………………………………2  Didn’t answer …………………….99 | | | Next section |
| **609** | What were the complications?  Multiple Response |  | Yes | No |  |
|  |  | Hemorrhage/vaginal bleeding | 1 | 0 |  |
|  |  | Incomplete MR | 1 | 0 |  |
|  |  | Infection | 1 | 0 |  |
|  |  | Headache | 1 | 0 |  |
|  |  | Vomiting | 1 | 0 |  |
|  |  | Fever | 1 | 0 |  |
|  |  | Abdominal pain | 1 | 0 |  |
|  |  | Irregular menstruation | 1 | 0 |  |
|  |  | Weakness | 1 | 0 |  |
|  |  | Others (Specify)…………………. 96 | | |  |
|  |  | Didn’t answer …………………….99 | | |  |
| **610** | Did seek any help for the complication (s)? | Yes ……………………………….1  No …………………………………2  Didn’t answer …………………….99 | | | 612 |
| **611** | From where you received help/services?  (Instruction: If the respondent says any name, probe if that is healthcare facility) | Home ………………………………1  Healthcare Facility (Specify) ……....2  Others (Specify)…………………. 96  Didn’t answer …………………….99 | | | Next section |
| **612** | (If you didn’t seek service), What was the reason for not seeking help / service?  Multiple Response |  | Yes | No |  |
|  |  | Did not feel the necessity | 1 | 0 |  |
|  |  | Did not know where/who to go | 1 | 0 |  |
|  |  | Did not know such care | 1 | 0 |  |
|  |  | Health center is far/Access to facility was/is difficult | 1 | 0 |  |
|  |  | Services were/are not available here | 1 | 0 |  |
|  |  | Did not like services provided in the facilities | 1 | 0 |  |
|  |  | Family/Husband did not allow | 1 | 0 |  |
|  |  | Did not have money | 1 | 0 |  |
|  |  | Nobody to accompany | 1 | 0 |  |
|  |  | Previous experience is not good | 1 | 0 |  |
|  |  | Concerned about being treated by a male doctor | 1 | 0 |  |
|  |  | Afraid to go health care center | 1 | 0 |  |
|  |  | Hadn’t time | 1 | 0 |  |
|  |  | Neighbor forbade to go | 1 | 0 |  |
|  |  | Health care center was far away from home | 1 | 0 |  |
|  |  | Others (Specify) …………………….96 | | |  |
|  |  | Didn’t Answer ………………………99 | | |  |

**Section 7: Sexually Transmitted Diseases**

| **No.** | **Questions and instructions** | **Coding categories** | | | **Skip** |
| --- | --- | --- | --- | --- | --- |
| **701** | There are some diseases that transmitted through sexual act/intercourse. Have you heard about of any of such diseases? | Yes ……………………………….1  No …………………………………2  Didn’t answer …………………….99 | | |  |
| **702** | Have you heard the name of HIV/AIDS? | Yes ……………………………….1  No …………………………………2 | | |  |
| **703** | Have you recently had any of these symptoms after coming here? (*read out one by one and mark all she mentioned*)  MULTIPLE RESPONSE |  | Yes | No | Next Section |
|  |  | Vaginal itching | 1 | 0 |  |
|  |  | Vaginal blisters or blisters in the genital area (the region covered by underwear) | 1 | 0 |  |
|  |  | Vaginal rash or rash in the genital area | 1 | 0 |  |
|  |  | Burning urination | 1 | 0 |  |
|  |  | Painful urination | 1 | 0 |  |
|  |  | Pain during intercourse | 1 | 0 |  |
|  |  | Bleeding or spotting between menstrual cycles | 1 | 0 |  |
|  |  | Painless ulcers on the vagina | 1 | 0 |  |
|  |  | Pelvic pain | 1 | 0 |  |
|  |  | Rectal pain, bleeding, or discharge (after receiving anal sex) | 1 | 0 |  |
|  |  | No symptoms | 1 | 0 |  |
|  |  | Never had physical relation | 1 | 0 |  |
|  |  | Others …………………………….96 | | |  |
|  |  | Not Applicable ……………………98 | | |  |
| **704** | Did you seek any treatment for any of the symptom you just mentioned? | Yes ……………………………….1  No …………………………………2  Didn’t answer …………………….99 | | | 7.6  Next Section |
| **705** | Where did you seek treatment?  (Instruction: If the respondent says any name, probe if that is healthcare facility) | Home ………………………………1  Healthcare Facility (Specify) ……...2  Others (Specify)…………………. 96  Didn’t answer …………………….99 | | |  |
| **706** | (If you didn’t seek treatment), What was the reason for not seeking treatment? |  | Yes | No |  |
|  |  | Did not feel the necessity | 1 | 0 |  |
|  |  | Did not know where/who to go | 1 | 0 |  |
|  |  | Did not know such care | 1 | 0 |  |
|  |  | Health center is far/Access to facility was/is difficult | 1 | 0 |  |
|  |  | Services were/are not available here | 1 | 0 |  |
|  |  | Did not like services provided in the facilities | 1 | 0 |  |
|  |  | Family/Husband did not allow | 1 | 0 |  |
|  |  | Did not have money | 1 | 0 |  |
|  |  | Nobody to accompany | 1 | 0 |  |
|  |  | Previous experience is not good | 1 | 0 |  |
|  |  | Concerned about being treated by a male doctor | 1 | 0 |  |
|  |  | Afraid to go health care center | 1 | 0 |  |
|  |  | Hadn’t time | 1 | 0 |  |
|  |  | Neighbor forbade to go | 1 | 0 |  |
|  |  | Health care center was far away from home | 1 | 0 |  |
|  |  | Others (Specify)……………………… 96 | | |  |
|  |  | Didn’t answer ………………………...99 | | |  |

**Section 8: Service utilization and barriers**

| **No.** | **Questions and instructions** | **Coding categories** | | | | | **Skip** |
| --- | --- | --- | --- | --- | --- | --- | --- |
| 801 | Have you ever received any SRH service (inclusive of any of the above or for other services) for yourself from any health worker or doctor or health center? | Yes ……………………………….1  No …………………………………2  Didn’t answer …………………….99 | | | | | 811  End |
| 802 | What services had you received?  MULTIPLE RESPONSE |  | Yes | | No | |  |
|  |  | Contraceptive method | 1 | | 0 | |  |
|  |  | Antenatal care | 1 | | 0 | |  |
|  |  | Delivery care | 1 | | 0 | |  |
|  |  | Postnatal care | 1 | | 0 | |  |
|  |  | MR/post abortion care | 1 | | 0 | |  |
|  |  | STI/STD | 1 | | 0 | |  |
|  |  | Others (Specify)………………………...96 | | | | |  |
|  |  | Did not answer………………………….99 | | | | |  |
| 803 | From who/where had you received services?  (Instruction: If the respondent says any name, probe if that is healthcare facility) | Home ………………………………1  Healthcare Facility (Specify) ……...2  Others (Specify)…………………. 96  Didn’t answer …………………….99 | | | | | End |
| 804 | Why did you choose that facility?  MULTIPLE RESPONSE |  | Yes | | No | |  |
|  |  | Close to my house | 1 | | 0 | |  |
|  |  | Suggested by husband | 1 | | 0 | |  |
|  |  | Suggested by other family member | 1 | | 0 | |  |
|  |  | Suggested by Neighbors | 1 | | 0 | |  |
|  |  | Recommended by health workers visited home | 1 | | 0 | |  |
|  |  | Recommended by Majhi | 1 | | 0 | |  |
|  |  | Doctor/ Nurse suggested | 1 | | 0 | |  |
|  |  | NGO suggested | 1 | | 0 | |  |
|  |  | Not Applicable | 1 | | 0 | |  |
|  |  | Others (Specify)………………………..96 | | | | |  |
|  |  | Did not answer………………………….99 | | | | |  |
| 805 | Now I want to know about your experience with the services you received.  How long did it take to go to the health facility? | \| Less than 15 minutes ……………….1   \| 15 to 30 minutes………………….2 \| \| --- \| \| 30 minutes to 1 hour…………..…3 \| \| 1 hour to 2 hour………………….4 \| \| more than 2 hours………………..5 \| \| Not Applicable…………………..6 \| \| \| --- \| --- \| --- \| --- \| --- \| --- \| | | | | |  |
| 806 | How did you go there? |  | Yes | | No | |  |
|  |  | By walking | 1 | | 0 | |  |
|  |  | By rickshaw/van | 1 | | 0 | |  |
|  |  | Ambulance | 1 | | 0 | |  |
|  |  | CNG | 1 | | 0 | |  |
|  |  | Tomtom | 1 | | 0 | |  |
|  |  | Bus | 1 | | 0 | |  |
|  |  | Hemorrhage/vaginal bleeding | 1 | | 0 | |  |
|  |  | Not Applicable | 1 | | 0 | |  |
|  |  | Others (Specify)……………………….96 | | | | |  |
|  |  | Did not answer………………………….99 | | | | |  |
| 807 | How long did you had to wait at the facility for getting the services (after entering to facility to receive service)?  *If they respond in hours please convert it in minutes* | Minutes \|__\|__\| | | | | |  |
| 808 | Did you feel comfortable enough to explain your problem and ask questions to the service provider? | Yes ………………………………….1  No …………………………………...2  Didn't answer……………………….. 99 | | | | | 8.10 |
| 809 | Why didn’t you feel comfortable?  MULTIPLE RESPONSE |  | Yes | | No | |  |
|  |  | Afraid of service provider | 1 | | 0 | |  |
|  |  | Didn’t understand language | 1 | | 0 | |  |
|  |  | Felt shy | 1 | | 0 | |  |
|  |  | There was no privacy | 1 | | 0 | |  |
|  |  | Service provider was male | 1 | | 0 | |  |
|  |  | Service provider behaved badly | 1 | | 0 | |  |
|  |  | Service provider didn’t ask | 1 | | 0 | |  |
|  |  | The center was very crowded | 1 | | 0 | |  |
|  |  | Environment was unfamiliar | 1 | | 0 | |  |
|  |  | There was no seating arrangement | 1 | | 0 | |  |
|  |  | Service provider was in rush and didn’t allow time | 1 | | 0 | |  |
|  |  | Others (Specify)…………………. 96 | | | | |  |
| 810 | What did you like about the facility you visited?  MULTIPLE RESPONSE |  | | Yes | | No | End |
|  |  | Clean Place | | 1 | | 0 |  |
|  |  | Good behavior of service providers | | 1 | | 0 |  |
|  |  | Close to home | | 1 | | 0 |  |
|  |  | Provided medicine | | 1 | | 0 |  |
|  |  | Easy to access | | 1 | | 0 |  |
|  |  | Providers understand my language | | 1 | | 0 |  |
|  |  | Female provider examined | | 1 | | 0 |  |
|  |  | Less waiting time | | 1 | | 0 |  |
|  |  | Maintain privacy | | 1 | | 0 |  |
|  |  | Good treatment | | 1 | | 0 |  |
|  |  | Others (Specify) …….……………96 | | | | |  |
|  |  | Not Applicable ………………...….97 | | | | |  |
|  |  | Did Not Answer …………….…....99 | | | | |  |
| 811 | What are the reasons that you did not receive services from any health facility/ health worker?  MULTIPLE RESPONSE |  | | Yes | | No |  |
|  |  | Did not feel the necessity | | 1 | | 0 |  |
|  |  | Did not know where/who to go | | 1 | | 0 |  |
|  |  | Did not know such care | | 1 | | 0 |  |
|  |  | Health center is far/Access to facility was/is difficult | | 1 | | 0 |  |
|  |  | Services were/are not available here | | 1 | | 0 |  |
|  |  | Did not like services provided in the facilities | | 1 | | 0 |  |
|  |  | Family/Husband did not allow | | 1 | | 0 |  |
|  |  | Did not have money | | 1 | | 0 |  |
|  |  | Nobody to accompany | | 1 | | 0 |  |
|  |  | Previous experience is not good | | 1 | | 0 |  |
|  |  | Concerned about being treated by a male doctor | | 1 | | 0 |  |
|  |  | Afraid to go health care center | | 1 | | 0 |  |
|  |  | Hadn’t time | | 1 | | 0 |  |
|  |  | Neighbor forbade to go | | 1 | | 0 |  |
|  |  | Health care center was far away from home | | 1 | | 0 |  |
|  |  | Others (Specify)………………………..96 | | | | |  |
|  |  | Did not answer………………………….99 | | | | |  |

**Do you think health facilities, should ask about exposure to violence (Domestic/GBV/IPV)? *Probe***
