# Supplementary material for: Situation analysis for delivering integrated comprehensive sexual and reproductive health services for displaced population of Kasaï, Democratic Republic of Congo: Protocol for a mixed method study
Source: PLoS One. 2020 Dec 21;15(12):e0242046. doi: 10.1371/journal.pone.0242046 (PMC7751877; doi:10.1371/journal.pone.0242046)
Supplement: S3 Annex — (DOCX) [file pone.0242046.s004.docx]

**Situation Analysis for Delivering Integrated Comprehensive SRHR Services for Kasaï displaced people, Democratic Republic of Congo**

**Facility Assessment**

| **Date: \|__\|__\|__\|__\|__\|__\|__\|__\|** | **Questionnaire Number: \|__\|__\|__\|__\|__\|__\|__\|__\|** |
| --- | --- |
| **Name of Facility: _______________________** | **Code: \|__\|__\|__\|__\|__\|__\|__\|__\|** |
| **Village/ Camp: ___________________** | **Code: \|__\|__\|__\|__\|__\|__\|__\|__\|** |
| **Health zone** | **Code: \|__\|__\|__\|__\|__\|__\|__\|__\|** |
| **Province : ___________________** | **Code: \|__\|__\|__\|__\|__\|__\|__\|__\|** |
| **Name of the Respondent** **___________________** | **Code: \|__\|__\|__\|__\|__\|__\|__\|__\|** |
| **Respondent designation** **___________________** | **Code: \|__\|__\|__\|** |

| **Interviewer Name** |  |  | **Code: \|__\|__\|__\|__\|__\|__\|** |
| --- | --- | --- | --- |

| **CONSENT** |
| --- |
| *Find the manager, the person in-charge of the facility, or the most senior health worker responsible for client services who is present at the facility. read the following greeting:*  *Good day! My name is _____________________. We are here on behalf of* ***Population and Health Research Institute*** *conducting a survey of health facilities to assist the government in knowing more about health services of displaced people in [****Province****].*  *Now I will read a statement explaining the study. Your facility was selected to participate in this study. We will be asking you questions about various health services. Information collected about your facility during this study may be used by the [MOH], organizations supporting services in your facility, and researchers, for planning service improvement or for conducting further studies of health services.*  *Neither your name nor the names of any other health workers who participate in this study will be included in the dataset or in any report; however, there is a small chance that any of these respondents may be identified later. Still, we are asking for your help to in order to collect this information. You may refuse to answer any question or choose to stop the interview at any time. However, we hope you will answer the questions, which will benefit the services you provide and the nation.*  *If there are questions for which someone else is the most appropriate person to provide the information, we would appreciate if you introduce us to that person to help us collect that information.*  *At this point, do you have any questions about the study? Do I have your agreement to proceed?*  ___________________________________________________________ \|__\|__\|__\|__\|2019  INTERVIEWER'S SIGNATURE INDICATING CONSENT OBTAINED. Date |

| Start time: |  | End time: |
| --- | --- | --- |

1. **General Information**

| **No** | **QUESTIONS** | **CODE** | **SKIP** |
| --- | --- | --- | --- |
| 101 | Types of the facility (*Please circle the appropriate one*)   \| 1. Referral hospital \| \| --- \| \| 2. Hospital \| \| 3. FP clinic \| \| 4. Health center/ Health Post \| \| 5. Mobile health clinic \| \| 6. Dispensary \| \| 9. Other (Specify__________________________________________________ \| |  |  |
| 102 | Management of the facility (*Please circle the appropriate one*)   \| 1. Government/public \| \| --- \| \| 2. National NGO \| \| 3. International NGO \| \| 4. Private-for-profit \| \| 5. Mission/Faith-based \| \| 6. FP association (IPPF affiliate) \| \| 9. Other \| |  |  |
| 103 | \| Source of external Financial/resource support if applicable \| \| --- \| \| A. Government \| \| B. National NGO \| \| C. International NGO \| \| D. Private donors \| \| E. No External support \| \| X. Other______________________ \| \| *Please circle all appropriate answers* \| |  |  |
|  | Number of days per week the facility is opened? (Please circle the appropriate one) |  |  |
| 104 | 1. One day a week |  |  |
|  | 2. Two days a week |  |  |
|  | 3. Three days a week |  |  |
|  | 4. Four days a week |  |  |
|  | 5. Five days a week |  |  |
|  | 6. Six days a week |  |  |
|  | 7. Seven days a week |  |  |
|  | 9. Other (specify)____________________ |  |  |
|  | *Please circle the appropriate one* |  |  |
| 105a | Open Hours: | |  |
|  | From \|__\|__\| : \|__\|__\| to \|__\|__\| : \|__\|__\| |  |  |
|  |  |  |  |
| 105b | Total Open hours : \|__\|__\| : \|__\|__\| |  |  |
|  |  |  |  |
| 106 | **Location** | |  |
|  | 1. Urban |  |  |
|  | 2. Rura |  |  |
| 107 | Estimated Number of outpatients per month : \|__\|__\|__\|__\|__\|__\|__\| | |  |
|  |  |  |  |
| 108 | Estimated Number of inpatients per month : \|__\|__\|__\|__\|__\|__\|__\| |  |  |
|  |  |  |  |

**2. Availability of sexual and reproductive health services**

| **No.** | **Name of the services** | **Code**  **1 = Yes**  **0 = Not available** | **Designation of the service provider/s**  **Code: 1, 2, 3, 4, 5, 6, 7, 8, 9, 97 *(for the details of this code follow the attached codebook)*** | **If the service is not available, then do you refer the patients?**  **Code**  **1 = Yes**  **0 = No** | **If yes, write the referred place/s**  ***(Most frequently referred)*** |
| --- | --- | --- | --- | --- | --- |
| 201 | ANC |  |  |  |  |
| 2011 | **Obstetric and foetal assessment** –   - Maternal weight - BP measurement - Oedema - Fundal height - Foetal heartbeat |  |  |  |  |
| 2012 | **Screening and tests**  Urinalysis  Hb estimation  Blood grouping and Rh typing  Testing for HIV, syphilis  Blood sugar  Ultrasonogram (referred cases for suspicion of low foetal growth |  |  |  |  |
| 2013 | **Identify & manage obstetric emergencies**  Pre/eclampsia  Ante-partum Haemorrhage  Abdominal pain  Premature rupture of membranes |  |  |  |  |
| 2014 | **Health education, advice, and counselling** (Nutrition, Complications of pregnancy / danger sign, Rest, Birth plan and emergency plan) |  |  |  |  |
| 2015 | Eclampsia Management |  |  |  |  |
| 2016 | PPH (Postpartum Hemorrhage) Management |  |  |  |  |
| 2017 | Maternal Immunization |  |  |  |  |
| 2018 | Delivery Care  Normal Vaginal Delivery  C-section  Deliveries that require vacuum extraction assistance |  |  |  |  |
| 2019 | Initial stabilization of obstetric emergency before referral |  |  |  |  |
| 2020 | Blood transfusion |  |  |  |  |
| 2021 | Essential new born care |  |  |  |  |
| 2022 | Post Natal Care |  |  |  |  |
| 2023 | Counselling on postnatal care, breastfeeding,  etc. |  |  |  |  |
| 2024 | Post-Natal clinical history (pain, fever,  haemorrhage) |  |  |  |  |
| 2025 | Identification and management of post-natal complications:  -Anaemia  -Puerperal psychosis |  |  |  |  |
| 2026 | Identification and management of obstetric complications:  -Haemorrhage  -Puerperal infection/sepsis |  |  |  |  |
| 2027 | Supply of Iron and Folic Acid |  |  |  |  |
| 2028 | Counselling and Provision of FP methods |  |  |  |  |
| 2029 | Family Planning |  |  |  |  |
| 2030 | FP services: Pill |  |  |  |  |
| 2031 | FP services: Male Condoms |  |  |  |  |
| 2032 | Emergency Contraception |  |  |  |  |
| 2033 | FP services: Injection |  |  |  |  |
| 2034 | FP services: Implant/Norplant |  |  |  |  |
| 2035 | FP services: IUD |  |  |  |  |
| 2036 | FP services: Vasectomy |  |  |  |  |
| 2037 | FP services: Tubectomy |  |  |  |  |
| 2038 | STD/RTI Management |  |  |  |  |
| 2039 | HIV AIDS Counseling |  |  |  |  |
| 2040 | HIV AIDS Testing |  |  |  |  |
| 2041 | Prevention of maternal –to-child transmission of HIV (PMTCT) *(one kind of medicine to stop transmitting HIV to the newborn)* |  |  |  |  |
| 2042 | Menstrual regulation (MR) |  |  |  |  |
| 2043 | Management of miscarriage and complications of abortions |  |  |  |  |
| 2044 | Clinical Management of Rape (CMR) |  |  |  |  |
| 2045 | Is there a written protocol/ SOP for provision of clinical management of rape available in the facility? |  |  |  |  |
| 2046 | Does the unit/department provide the following elements of post-rape care/care to survivors of sexual violence? |  |  |  |  |
| 2047 | Emergency Contraception |  |  |  |  |
| 2048 | HIV post-exposure prophylaxis |  |  |  |  |
| 2049 | STI prophylaxis/ presumptive treatment |  |  |  |  |
| 2050 | Menstrual regulation |  |  |  |  |
| 2051 | Psychological support, crisis counseling, psychological first aid |  |  |  |  |
| 2052 | Do you have a referral directory or referral pathway with the names and contact details of other organizations/services that respond to cases of violence? |  |  |  |  |
| 2053 | Adolescent friendly health services (counseling on Sexuality education/ sexual health care) |  |  |  |  |
| 2054 | Adolescent friendly health services (counseling on FP services) |  |  |  |  |
| 2055 | Adolescent friendly health services (counseling on HIV services) |  |  |  |  |
| 2056 | Others |  |  |  |  |

202 If you don’t provide the SRHR services, please mention the reason for not providing the SRHR services.

_______________________________________________________________________________

_______________________________________________________________________________

_______________________________________________________________________________

**3. Human Resources: Key Staff information**

| **No.** | **Categories** | **No of staff** | **Number working Fulltime** |
| --- | --- | --- | --- |
| 301 | Gynecologists / Gynae Consultant |  |  |
| 302 | Anesthesiologist |  |  |
| 303 | Residential medical officer (RMO) |  |  |
| 304 | Medical officer |  |  |
| 305 | Medical assistant |  |  |
| 306 | Health inspector |  |  |
| 307 | Nursing supervisor |  |  |
| 308 | Nurse |  |  |
| 309 | Midwife |  |  |
| 310 | Trained birth attendants (TBAs) |  |  |
| 311 | Family welfare assistant (FWA) |  |  |
| 312 | Community health worker (CHW) |  |  |
| 313 | Psycho-social counsellor |  |  |
| 314 | Laboratory technicians/ medical technologist |  |  |
| 315 | Ward boy |  |  |
| 316 | Driver |  |  |
| 317 | Cleaner |  |  |
| 997. | Others (specify) |  |  |

**4. Training**

**If any of your staff received any of the following training:**

| **Serial No.** | **Types of Training (RECEIVED IN THE PAST Three MONTHS)** | **Code**  **1 = Yes**  **0 = No** | **Who received it (Doctor, Nurse, Midwifes etc?) Code: 1 – 20, 97 *(for the details of this code follow the attached codebook)*** |
| --- | --- | --- | --- |
| 401 | Helping Babies Breathe |  |  |
| 402 | Emergency Response |  |  |
| 403 | Helping Mothers Survive |  |  |
| 404 | Domestic violence |  |  |
| 405 | Clinical Management of Rape (CMR)  training on |  |  |
| 406 | Psychological First Aid or Psychosocial Support |  |  |

| 407 | Responding domestic violence? |  |  |
| --- | --- | --- | --- |
| 408 | Responding to sexual violence ? |  |  |

|  | OGSB Training - FP (Long Acting & Permanent) |  |  |
| --- | --- | --- | --- |
|  | EmONC |  |  |
|  | OGSB Training - Infection Prevention Control |  |  |
|  | OGSB Training – RTI & STI |  |  |
|  | OGSB Training – Management & Care of Maternal & Child Nutrition |  |  |
|  | ANC Package |  |  |
|  | MR & PAC |  |  |
|  | Orientation on community distribution of misoprostol |  |  |
|  | MISP Training for Coordinator |  |  |
|  | OGSB Training – Safe Delivery Practice |  |  |
|  | Labor Room Protocol |  |  |
|  | Cervical Cancer Screening |  |  |
|  | FP Counselling |  |  |
|  | IUD |  |  |
|  | Implant |  |  |
|  | PPIUD |  |  |
|  | Others |  |  |

| **No** | **QUESTIONS** | **CODE** | **SKIP** |
| --- | --- | --- | --- |
| 409 | Do you think is there any other training that you/your staff need to receive?   \| 1. Yes \| \| --- \| \| 2. No \| |  | If No (2) Go to 501 |
| 410 | If Yes, specify ____________________________________ |  |  |
|  |  |  |  |

**5. Information on total number of services**

| **No** | **QUESTIONS** | **CODE** | **SKIP** |
| --- | --- | --- | --- |
| 501 | Average total number of Outpatient served in a day? | \|__\|__\|__\|__\| |  |
| 502 | Total number of inpatients served in a month? (Last month/3 month average) | \|__\|__\|__\|__\| |  |

503. Information on total number of services

| **No.** | **Services and Quantity** | **April 2019** | **May 2019** | **June 2019** |
| --- | --- | --- | --- | --- |
| 5031 | No. of total patients served |  |  |  |
| 5032 | No. of ANC |  |  |  |
| 5033 | Eclampsia Management |  |  |  |
| 5034 | PPH (Postpartum Hemorrhage) Management |  |  |  |
| 5035 | Total delivery |  |  |  |
| 5036 | Normal Delivery |  |  |  |
| 5037 | C section |  |  |  |
| 5038 | No. of PNC |  |  |  |
| 5038 | Blood Transfusion |  |  |  |
| 5039 | No. of FP counseling |  |  |  |
| 5040 | FP services: Pill |  |  |  |
| 5041 | FP services: Condom |  |  |  |
| 5042 | FP services: Injection |  |  |  |
| 5043 | FP services: Implant/Norplant |  |  |  |
| 5044 | FP services: IUD |  |  |  |
| 5045 | FP services: Vasectomy |  |  |  |
| 5046 | FP services: Tubectomy |  |  |  |
| 5047 | No. of STD/RTI screening |  |  |  |
| 5048 | No. of STD/RTI treatment |  |  |  |
| 5049 | No of HIV/AIDS counselling |  |  |  |
| 5050 | No of HIV/AIDS screening |  |  |  |
| 5051 | No. of MR service |  |  |  |
| 5052 | No. of management of miscarriage and complications of abortions |  |  |  |
| 5053 | No of Post rape treatment:  - Emergency contraception |  |  |  |
| 5054 | No of Post rape treatment: PEP for HIV |  |  |  |
| 5055 | No of Post rape treatment: STI prophylaxis/presumptive treatment |  |  |  |
| 5056 | No. of adolescent health service |  |  |  |
| 5057 | Total no. of referred patients |  |  |  |
| 5058 | Other (Specify |  |  |  |

**6. Availability of ambulance service**

- - 1. If yes, then how many do you have?

| **No** | **QUESTIONS** | **CODE** | **SKIP** |
| --- | --- | --- | --- |
| 601 | **Is there any ambulance services in your facility?** | |  |
|  | 1. Yes |  |  |
|  | 2. No |  |  |
| 602 | If yes, then how many do you have? | \|__\|__\| |  |
|  | **If No, do you have access to other’s (private/NGO/Govt) ambulance services that you could use ?** |  | If No, Go to 605 |
| 603 | 1. Yes |  |  |
|  | 2. No |  |  |
|  |  |  |  |
| 604 | If Yes, specify______________________________ |  |  |
|  |  |  |  |
|  | When do you refer patients, how do you transport them to the referred  place? (Mode of transportation) |  |  |
| 605 | ____________________________________________________ |  |  |
|  |  |  |  |

**7. Structure of the facility /Availability of rooms (related to SRH)**

| **No.** | **Name of department/room** | **Code**  **1 = Yes**  **0 = Not available** | **Remark** |
| --- | --- | --- | --- |
| 701 | Emergency room |  |  |
| 702 | Consultant’s room |  |  |
| 703 | Counselling room |  |  |
| 704 | Patient’s waiting place |  |  |
| 705 | ANC/PNC room |  |  |
| 706 | Labor Room |  |  |
| 707 | Labor Ward |  |  |
| 708 | Women Ward |  |  |
| 709 | VIA/PAC room |  |  |
| 710 | Room for USG |  |  |
| 711 | Laboratory for lab tests |  |  |
| 712 | Room for blood transfusion |  |  |
| 713 | Maternity OPD |  |  |
| 714 | Breast feeding corner in OPD |  |  |
| 715 | General Operation Theatre |  |  |
| 716 | Dispensary |  |  |
| 797 | Other (Specify) |  |  |

720 Number of maternity beds at the facility

721 Number of beds occupied by the patients on average

722 Is there a space (for example, a room or area) available for private and confidential consultation (that is that ensures survivors from violence cannot be seen or heard from outside)?

0- No space

1- Yes, there is only a space that allows for visual privacy

2- Yes, there is only a space that allows for auditory privacy,

3- Yes, there is a space that allows fpr both visual and audiotry privacy,

4- don’t know)

**8 Equipment, drugs and supplies**

| No | KIT Number & Name | Received? Yes = 1  No = 0 | If yes, How many? (Boxes) | If No, Then Why? | If not a UNFPA KIT receiver then how do you procure/ source those drugs and supplies |
| --- | --- | --- | --- | --- | --- |
| 801 | Kit 1: Condoms |  |  |  |  |
| 802 | Kit 2: Clean Delivery, Individual |  |  |  |  |
| 803 | Kit 3: Post Rape Treatment |  |  |  |  |
| 804 | Kit 4 Oral And Injectable Contraception |  |  |  |  |
| 805 | Kit 5: Treatment Of Sexually Transmitted Infections |  |  |  |  |
| 806 | Kit 6: Clinical Delivery Assistance |  |  |  |  |
| 807 | Kit 7: Intrauterine Device |  |  |  |  |
| 808 | Kit 8: Management Of Miscarriage And Complications Of Abortion |  |  |  |  |
| 809 | Kit 9: Suture Of Tears (Cervical & Vaginal Examination) |  |  |  |  |
| 810 | Kit 10: Vacuum Extraction Delivery |  |  |  |  |
| 811 | Kit 11: Referral Level Kit For Reproductive Health |  |  |  |  |
| 812 | Kit 12 Blood Transfusion |  |  |  |  |

**9.Infrastructure**: Standard precautions for infection prevention (ref: SARA reference manual of WHO)

| **No.** | **Activities** | **Code**  **1 = Yes**  **0 = Not available** | **Remarks *(How?)*** |
| --- | --- | --- | --- |
| 901 | Appropriate storage of sharp object wastes (sharps box/container) |  |  |
| 902 | Appropriate storage of infectious waste (waste receptacle with lid and plastic bin liner) |  |  |
| 903 | Safe final disposal of sharp objects |  |  |
| 904 | Safe final disposal of infectious wastes |  |  |

**ANNEXURE**

Code Book:

- 1. Health Service Information on SRHR Designation of the service provider/s Code: 1 – 20, 97 *(for the details please follow below mentioned list)*

| 1 | Gynecologists / Gynae Consultant | 11 | Midwife |
| --- | --- | --- | --- |
| 2 | Anesthesiologist | 12 | Trained Birth Attendants (TBA) |
| 3 | Residential Medical Officer (RMO) | 13 | Other community health workers (FWA) |
| 4 | Medical Officer | 14 | CHCP |
| 5 | Medical Assistant | 15 | Counsellor |
| 6 | SACMO | 16 | Laboratory technicians/ Medical Technologist |
| 7 | Health Inspector | 17 | Ward Boy |
| 8 | Nursing Supervisor | 18 | Driver |
| 9 | Senior Staff Nurse | 19 | Cleaner |
| 10 | Staff Nurse | 97 | Other (specify) |
