# Supplementary material for: Situation analysis for delivering integrated comprehensive sexual and reproductive health services for displaced population of Kasaï, Democratic Republic of Congo: Protocol for a mixed method study
Source: PLoS One. 2020 Dec 21;15(12):e0242046. doi: 10.1371/journal.pone.0242046 (PMC7751877; doi:10.1371/journal.pone.0242046)
Supplement: S4 Annex — (PDF) [file pone.0242046.s005.pdf]

Situation Analysis for Delivering Integrated Comprehensive SRHR Services for Kasaï displaced people, Democratic Republic of Congo

Facility Assessment

**Date:**	|__|__|__|__|__|__|__|__|__|	**Questionnaire Number:**	|__|__|__|__|__|__|__|__|__|
**Name of Facility:** ______________________		**Code:**	|__|__|__|__|__|__|__|__|__|
**Village/ Camp:** __________________		**Code:**	|__|__|__|__|__|__|__|__|__|
**Health zone**		**Code:**	|__|__|__|__|__|__|__|__|__|
**Province :** __________________		**Code:**	|__|__|__|__|__|__|__|__|__|
**Name of the Respondent** __________________		**Code:**	|__|__|__|__|__|__|__|__|__|
**Respondent designation** __________________		**Code:**	|__|__|__|

Interviewer Name **Code:** |__|__|__|__|__|__|__|

CONSENT

Find the manager, the person in-charge of the facility, or the most senior health worker responsible for client services who is present at the facility. read the following greeting:

Good day! My name is ____________________. We are here on behalf of ***Population and Health Research Institute*** *conducting a survey of health facilities to assist the government in knowing more about health services of displaced people in [****Province****].*

Now I will read a statement explaining the study. Your facility was selected to participate in this study. We will be asking you questions about various health services. Information collected about your facility during this study may be used by the [MOH], organizations supporting services in your facility, and researchers, for planning service improvement or for conducting further studies of health services.

Neither your name nor the names of any other health workers who participate in this study will be included in the dataset or in any report; however, there is a small chance that any of these respondents may be identified later. Still, we are asking for your help to in order to collect this information. You may refuse to answer any question or choose to stop the interview at any time. However, we hope you will answer the questions, which will benefit the services you provide and the nation.

If there are questions for which someone else is the most appropriate person to provide the information, we would appreciate if you introduce us to that person to help us collect that information.

At this point, do you have any questions about the study? Do I have your agreement to proceed?

____ |__|__|__|__|2019
INTERVIEWER'S SIGNATURE INDICATING CONSENT OBTAINED. Date

Start time: End time:

1. General Information

No	QUESTIONS	CODE	SKIP
101	Types of the facility (*Please circle the appropriate one*)<br>1. Referral hospital<br>2. Hospital<br>3. FP clinic<br>4. Health center/ Health Post<br>5. Mobile health clinic<br>6. Dispensary<br>9. Other<br>(Specify___	☐	
102	Management of the facility (*Please circle the appropriate one*)<br>1. Government/public<br>2. National NGO<br>3. International NGO<br>4. Private-for-profit<br>5. Mission/Faith-based<br>6. FP association (IPPF affiliate)<br>9. Other	☐	
103	Source of external Financial/resource support if applicable<br>A. Government<br>B. National NGO<br>C. International NGO<br>D. Private donors<br>E. No External support<br>X. Other____________________<br>*Please circle all appropriate answers*		
104	Number of days per week the facility is opened? (Please circle the appropriate one)<br>1. One day a week<br>2. Two days a week<br>3. Three days a week<br>4. Four days a week<br>5. Five days a week<br>6. Six days a week<br>7. Seven days a week<br>9. Other (specify)___________________<br>*Please circle the appropriate one*	☐	
105a	Open Hours:<br>From |__|__| : |__|__| to |__|__| : |__|__|		
105b	Total Open hours : |__|__| : |__|__|		
106	**Location**<br>1. Urban<br>2. Rura	☐	
107	Estimated Number of outpatients per month : |__|__|__|__|__|__|__|		
108	Estimated Number of inpatients per month : |__|__|__|__|__|__|__|		

2. Availability of sexual and reproductive health services

**No.**	**Name of the services**	**Code**<br><br>**1 = Yes**<br>**0 = Not available**	**Designation of the service provider/s**<br>**Code: 1, 2, 3, 4, 5, 6, 7, 8, 9, 97** ***(for the details of this code follow the attached codebook)***	**If the service is not available, then do you refer the patients?**<br>**Code**<br>**1 = Yes**<br>**0 = No**	**If yes, write the referred place/s**<br>***(Most frequently referred)***
201	ANC				
2011	**Obstetric and foetal assessment** –<br>• Maternal weight<br>• BP measurement<br>• Oedema<br>• Fundal height<br>• Foetal heartbeat				
2012	**Screening and tests**<br>Urinalysis<br>Hb estimation<br>Blood grouping and Rh typing<br>Testing for HIV, syphilis<br>Blood sugar<br>Ultrasonogram (referred cases for suspicion of low foetal growth				
2013	**Identify & manage obstetric emergencies**<br>Pre/eclampsia<br>Ante-partum Haemorrhage<br>Abdominal pain<br>Premature rupture of membranes				
2014	**Health education, advice, and counselling** (Nutrition, Complications of pregnancy / danger sign, Rest, Birth plan and emergency plan)				
2015	Eclampsia Management				

No.	Name of the services	Code<br><br>1 = Yes<br><br>0 = Not available	Designation of the service provider/s<br><br>Code: 1, 2, 3, 4, 5, 6, 7, 8, 9, 97 *(for the details of this code follow the attached codebook)*	If the service is not available, then do you refer the patients?<br><br>Code<br><br>1 = Yes<br>0 = No	If yes, write the referred place/s<br><br>*(Most frequently referred)*
2016	PPH (Postpartum Hemorrhage) Management				
2017	Maternal Immunization				
2018	Delivery Care<br>Normal Vaginal Delivery<br>C-section<br>Deliveries that require vacuum extraction assistance				
2019	Initial stabilization of obstetric emergency before referral				
2020	Blood transfusion				
2021	Essential new born care				
2022	Post Natal Care				
2023	Counselling on postnatal care, breastfeeding,<br>etc.				
2024	Post-Natal clinical history (pain, fever, haemorrhage)				
2025	Identification and management of post-natal complications:<br>-Anaemia<br>-Puerperal psychosis				
2026	Identification and management of obstetric complications:<br>-Haemorrhage<br>-Puerperal infection/sepsis				
2027	Supply of Iron and Folic Acid				
2028	Counselling and Provision of FP methods				
2029	Family Planning				
2030	FP services: Pill				
2031	FP services: Male Condoms				

No.	Name of the services	Code<br><br>1 = Yes<br>0 = Not available	Designation of the service provider/s<br>Code: 1, 2, 3, 4, 5, 6, 7, 8, 9, 97 *(for the details of this code follow the attached codebook)*	If the service is not available, then do you refer the patients?<br>Code<br>1 = Yes<br>0 = No	If yes, write the referred place/s<br>*(Most frequently referred)*
2032	Emergency Contraception				
2033	FP services: Injection				
2034	FP services: Implant/Norplant				
2035	FP services: IUD				
2036	FP services: Vasectomy				
2037	FP services: Tubectomy				
2038	STD/RTI Management				
2039	HIV AIDS Counseling				
2040	HIV AIDS Testing				
2041	Prevention of maternal –to-child transmission of HIV (PMTCT) *(one kind of medicine to stop transmitting HIV to the newborn)*				
2042	Menstrual regulation (MR)				
2043	Management of miscarriage and complications of abortions				
2044	Clinical Management of Rape (CMR)				
2045	Is there a written protocol/ SOP for provision of clinical management of rape available in the facility?				
2046	Does the unit/department provide the following elements of post-rape care/care to survivors of sexual violence?				
2047	Emergency Contraception				
2048	HIV post-exposure prophylaxis				
2049	STI prophylaxis/ presumptive treatment				
2050	Menstrual regulation				

No.	Name of the services	Code<br><br>1 = Yes<br><br>0 = Not available	Designation of the service provider/s<br><br>Code: 1, 2, 3, 4, 5, 6, 7, 8, 9, 97 *(for the details of this code follow the attached codebook)*	If the service is not available, then do you refer the patients?<br><br>Code<br><br>1 = Yes<br>0 = No	If yes, write the referred place/s<br><br>*(Most frequently referred)*
2051	Psychological support, crisis counseling, psychological first aid				
2052	Do you have a referral directory or referral pathway with the names and contact details of other organizations/services that respond to cases of violence?				
2053	Adolescent friendly health services (counseling on Sexuality education/ sexual health care)				
2054	Adolescent friendly health services (counseling on FP services)				
2055	Adolescent friendly health services (counseling on HIV services)				
2056	Others				

202 If you don’t provide the SRHR services, please mention the reason for not providing the SRHR services.

____

____

____

3. Human Resources: Key Staff information

No.	Categories	No of staff	Number working Fulltime
301	Gynecologists / Gynae Consultant		
302	Anesthesiologist		
303	Residential medical officer (RMO)		
304	Medical officer		
305	Medical assistant		
306	Health inspector		
307	Nursing supervisor		
308	Nurse		
309	Midwife		
310	Trained birth attendants (TBAs)		
311	Family welfare assistant (FWA)		
312	Community health worker (CHW)		
313	Psycho-social counsellor		
314	Laboratory technicians/ medical technologist		
315	Ward boy		
316	Driver		
317	Cleaner		
997.	Others (specify)		

4. Training
If any of your staff received any of the following training:

Serial No.	Types of Training (RECEIVED IN THE PAST Three MONTHS)	Code<br>1 = Yes<br>0 = No	Who received it (Doctor, Nurse, Midwifes etc?) Code: 1 – 20, 97 *(for the details of this code follow the attached codebook)*
401	Helping Babies Breathe		
402	Emergency Response		
403	Helping Mothers Survive		
404	Domestic violence		
405	Clinical Management of Rape (CMR) training on		
406	Psychological First Aid or Psychosocial Support		
407	Responding domestic violence?		
408	Responding to sexual violence ?		
1.	OGSB Training - FP (Long Acting & Permanent)		
2.	EmONC		
3.	OGSB Training - Infection Prevention Control		
4.	OGSB Training – RTI & STI		
5.	OGSB Training – Management & Care of Maternal & Child Nutrition		
6.	ANC Package		
7.	MR & PAC		
8.	Orientation on community distribution of misoprostol		
9.	MISP Training for Coordinator		
10.	OGSB Training – Safe Delivery Practice		
11.	Labor Room Protocol		
12.	Cervical Cancer Screening		

13.	FP Counselling		
14.	IUD		
15.	Implant		
16.	PPIUD		
17.	Others		

No	QUESTIONS	CODE	SKIP
409	Do you think is there any other training that you/your staff need to receive?<br>1. Yes<br>2. No	☐	If No (2) Go to 501
410	If Yes, specify ___________________________________		

5. Information on total number of services

No	QUESTIONS	CODE	SKIP
501	Average total number of Outpatient served in a day?	|__|__|__|__|	
502	Total number of inpatients served in a month? (Last month/3 month average)	|__|__|__|__|	

503. Information on total number of services

No.	Services and Quantity	April 2019	May 2019	June 2019
5031	No. of total patients served			
5032	No. of ANC			
5033	Eclampsia Management			
5034	PPH (Postpartum Hemorrhage) Management			
5035	Total delivery			
5036	Normal Delivery			
5037	C section			
5038	No. of PNC			
5038	Blood Transfusion			
5039	No. of FP counseling			
5040	FP services: Pill			
5041	FP services: Condom			
5042	FP services: Injection			
5043	FP services: Implant/Norplant			
5044	FP services: IUD			
5045	FP services: Vasectomy			
5046	FP services: Tubectomy			
5047	No. of STD/RTI screening			
5048	No. of STD/RTI treatment			
5049	No of HIV/AIDS counselling			

No.	Services and Quantity	April 2019	May 2019	June 2019
5050	No of HIV/AIDS screening			
5051	No. of MR service			
5052	No. of management of miscarriage and complications of abortions			
5053	No of Post rape treatment:<br>- Emergency contraception			
5054	No of Post rape treatment: PEP for HIV			
5055	No of Post rape treatment: STI prophylaxis/presumptive treatment			
5056	No. of adolescent health service			
5057	Total no. of referred patients			
5058	Other (Specify			

6. Availability of ambulance service

2.5.1 If yes, then how many do you have? ☐

No	QUESTIONS	CODE	SKIP
601	**Is there any ambulance services in your facility?**<br>1. Yes<br>2. No	☐	
602	If yes, then how many do you have?	|__|__|	
603	**If No, do you have access to other's (private/NGO/Govt) ambulance services that you could use ?**<br>1. Yes<br>2. No	☐	If No, Go to 605
604	If Yes, specify_______________________________		
605	When do you refer patients, how do you transport them to the referred place? (Mode of transportation)<br>____		

7. Structure of the facility /Availability of rooms (related to SRH)

No.	Name of department/room	Code<br>1 = Yes<br>0 = Not available	Remark
701	Emergency room		
702	Consultant's room		
703	Counselling room		
704	Patient's waiting place		
705	ANC/PNC room		
706	Labor Room		
707	Labor Ward		
708	Women Ward		

No.	Name of department/room	Code<br>1 = Yes<br>0 = Not available	Remark
709	VIA/PAC room		
710	Room for USG		
711	Laboratory for lab tests		
712	Room for blood transfusion		
713	Maternity OPD		
714	Breast feeding corner in OPD		
715	General Operation Theatre		
716	Dispensary		
797	Other (Specify)		

720 Number of maternity beds at the facility

721 Number of beds occupied by the patients on average

722 Is there a space (for example, a room or area) available for private and confidential consultation (that is that ensures survivors from violence cannot be seen or heard from outside)?
0- No space
1- Yes, there is only a space that allows for visual privacy
2- Yes, there is only a space that allows for auditory privacy,
3- Yes, there is a space that allows fpr both visual and audiotry privacy,
4- don't know)

8 Equipment, drugs and supplies

No	KIT Number & Name	Received?<br>Yes = 1<br>No = 0	If yes, How many? (Boxes)	If No, Then Why?	If not a UNFPA KIT receiver then how do you procure/ source those drugs and supplies
801	Kit 1: Condoms				
802	Kit 2: Clean Delivery, Individual				
803	Kit 3: Post Rape Treatment				
804	Kit 4 Oral And Injectable Contraception				
805	Kit 5: Treatment Of Sexually Transmitted Infections				
806	Kit 6: Clinical Delivery Assistance				

807	Kit 7: Intrauterine Device				
808	Kit 8: Management Of Miscarriage And Complications Of Abortion				
809	Kit 9: Suture Of Tears (Cervical & Vaginal Examination)				
810	Kit 10: Vacuum Extraction Delivery				
811	Kit 11: Referral Level Kit For Reproductive Health				
812	Kit 12 Blood Transfusion				

9.Infrastructure: Standard precautions for infection prevention (ref: SARA reference manual of WHO)

**No.**	**Activities**	**Code**<br>**1 = Yes**<br>**0 = Not available**	**Remarks *(How?)***
901	Appropriate storage of sharp object wastes (sharps box/container)		
902	Appropriate storage of infectious waste (waste receptacle with lid and plastic bin liner)		
903	Safe final disposal of sharp objects		
904	Safe final disposal of infectious wastes		

ANNEXURE

Code Book:

2.1 Health Service Information on SRHR Designation of the service provider/s Code: 1 – 20, 97 *(for the details please follow below mentioned list)*

1 Gynecologists / Gynae Consultant
2 Anesthesiologist
3 Residential Medical Officer (RMO)
4 Medical Officer
5 Medical Assistant
6 SACMO
7 Health Inspector
8 Nursing Supervisor
9 Senior Staff Nurse
10 Staff Nurse
11 Midwife
12 Trained Birth Attendants (TBA)
13 Other community health workers (FWA)
14 CHCP
15 Counsellor
16 Laboratory technicians/ Medical Technologist
17 Ward Boy
18 Driver
19 Cleaner
97 Other (specify)
