# Supplementary material for: Situation analysis for delivering integrated comprehensive sexual and reproductive health services for displaced population of Kasaï, Democratic Republic of Congo: Protocol for a mixed method study
Source: PLoS One. 2020 Dec 21;15(12):e0242046. doi: 10.1371/journal.pone.0242046 (PMC7751877; doi:10.1371/journal.pone.0242046)
Supplement: Attachment — Submitted filename: comments to authors.docx [file pone.0242046.s006.docx]

Dear authors,

The protocol for the situation analysis of SRHS for displaces population of Kasaï is an interesting topic that deserves full assessment to improve such health care delivery for displaced women. From the description this project is very ambitious and a huge amount of work will be required to pull this off, I’m assuming that the authors have sufficient time, human and financial resources to fulfil all the work.

The manuscript needs substantial revision for the English language, the manuscript should be edited by an English native speaker.

My concern in general is that this situation analysis concerns SHR health care delivery. However, from the protocol it was not very clear if the situation analysis of health care facilities that will be visited, if they were specifically set-up in these camps to deliver health care for the displaces population? Or if staff has received specific training for the care of post-conflict, displaced individuals? From reading the manuscript, the feeling is that it is a general SHR assessment of SHR rather than SHR assessment for displaced people. It seems that the health care facilities that will be visited and audited are the ones that are traditionally there to provide healthcare to the resident population. Then the outcome could be biased because of course these type of healthcare facilities will not be prepared to deliver post-traumatic healthcare.

Another concern is the focus of the study, data on SHR delivery will be collected from a large variety of sources, which is an advantage for triangulation, however the questionnaires of Qualitative research are very exhaustive collecting insights on SHR delivery for men, women, adolescent boys and girls. From the introduction I understood that the target study population was displaced women, while the Qualitative study is trying to gather insight on SHR delivery in general, this related back to my first concern. Do you want to know about SHR delivery in general? Or specifically for the displaced female population?

It is not explained in the text why you are sampling women and young girls from 12-49 years of age for the women questionnaire while the WHO definition of women of reproductive age is 15-49 years of age.

Lastly, my major concern is about the sampling of households and women for the community based survey. In the methods you describe that you will select from an updated list of villages/camps. Already in the introduction it is not well described if the displaced are living in villages (permanent housing) or in camps (temporary housing). The description of the context with a bit more detail will help to understand the methodology used. Later on in the expected challenges section you describe that the location and the ability to identify and recruit the target populations could be a challenge. The mitigation plan is to involve leaders and NGO’s, but my concern is how will you be able to apply the random sampling strategy if this housing list, village list, camp list are not available?

*Specific comments on the manuscript*

Abbreviations should be written in full the first time that it is used, not after (Also in the abstract)

Introduction: “ The region had the highest fertility (8 children per women versus the national average of 6.6 children per woman); significantly low modern contraceptive prevalence (6%) and **relatively low unmet needs for FP.”** This part of the sentence doesn’t make sense, the proportion relates to unmet needs? Or the needs that were met?

Study design: the first paragraph is quite confusing while reading. combining community-based survey with standard assessment does not reflect mixed method. I suggest to break this into multiple phrases. And actually your study design is 3 surveys: 1) community based survey (QUANT); 2) health facility assessment (QUANT) and 3) Key informant interviews and FGD (QUAL). Bringing this structure to the manuscript would much more comprehensive for the reader.

Participants: “the study will target different population” should be ‘target different study subjects’

You should describe better which study population will be targeted for which survey or questionnaire

Data sources:

- Will you recruit 500 households, or 500 women and young girls? The way you describe, more than 1 women can live in a household. Therefore the number of households in unknown, and you will recruit until you have reached the minimal sample size = 500 women and young girls
- “In addition, to the household and women questionnaire, a health facility assessment will be

administered. The health facility assessment will employ a health facility quantitative survey coupled with a facility audit.” This is very confusing as a transition sentence, it is as if you are going to administer this assessment to the same study population. You can separate these two surveys.

- “In each selected health facility, an audit, a provider’s interview, a **non-participatory observation and client exit interview** will be conducted.” The two activities in bolt, I have serious concern about this, due to confidentiality reasons. Especially since it relates to SRH, this might be very uncomfortable for the study subjects. You don’t mention it later on in the protocol, is this maintained or not? Personally I don’t agree for these methods for SRH.
- “A total sample of 60 providers in each site will be interviewed”; this is confusing as later on you say you will recruit 4 health care providers per site?
- The questionnaire for provider interview was not provided, you say you will assess “experience and perceptions” I’m assuming this is a quantitative questionnaire, therefore perception cannot be assessed, but rather attitudes and practices, like a (Knowledge, attitudes and practices) KAP design.
- Audit of the facility: read my concern on assessment of facilities in general? Or specifically targeted to displaced population?
- First time you mention FGD, it is not clear if men and women will be interviewed separately, you explain later on in the text that you will, but you should be clear from the start that this is a delicate subject, so women and men should not be mixed in the FGD.
- You should mention that FDGs are conducted until saturation of information is reached. It is highly doubtful that with the sample size of 12FGD you will reach this saturation. Especially when you split up as you describe. 4 FGD per province, 2 in men and 2 in women, and then from the two, you split into 1 in adults and 1 in youth. This means you only have 1 FGD per age group per sex. Re-consider the minimum sample size if you want to maintain this methodology.

Ethical considerations

You will take informed verbal consent for everybody?

I agree for the qualitative research, you can maintain verbal consent.

I disagree for the quantitative data. Especially in the health care facilities, providers are nurses and doctors, they should be able to sign, written informed consent can be asked here.

For the household survey written informed consent would be preferable, with finger print if respondents cannot read and write, however, verbal consent can justified. This justification should be mentioned.
